# Evaluation of physical and chemical modifications to drug reservoirs for stimuli-responsive microneedles

**DOI:** 10.1007/s13346-024-01737-0

**Published:** 2024-11-20

**Authors:** Luchi Li, Qonita Kurnia Anjani, Aaron R. J. Hutton, Mingshan Li, Akmal Hidayat Bin Sabri, Lalitkumar Vora, Yara A. Naser, Yushi Tao, Helen O. McCarthy, Ryan F. Donnelly

**Affiliations:** 1 School of Pharmacy, Medical Biology Centre, Queens University Belfast, 97 Lisburn Road, BT9 7BL Belfast, United Kingdom; 2 School of Pharmacy and Pharmaceutical Sciences, Ulster University, Coleraine, United Kingdom Block Y, 1SA, Cromore Rd, BT52 1SA

**Keywords:** Hydrogel-forming microneedle arrays, Iontophoresis, Heat-assisted drug delivery technology, Lyophilised reservoir, Effervescent reservoir

## Abstract

Hydrogel-forming microneedle (MN) arrays are minimally-invasive devices that can penetrate the *stratum corneum*, the main barrier to topical drug application, without causing pain. However, drug delivery using hydrogel-forming MN arrays tends to be relatively slow compared to rapid drug delivery using conventional needles and syringes. Therefore, in this work, for the first time, different physical and chemical delivery enhancement methods were employed in combination with PVA-based hydrogel-forming MN arrays. Using a model drug, ibuprofen (IBU) sodium, the designed systems were assessed in terms of the extent of transdermal delivery. Iontophoresis (ITP) and heat-assisted drug delivery technology were investigated as physical permeation enhancement techniques. Ex vivo studies demonstrated that the ITP (0.5 mA/cm^2^)-mediated combination strategy significantly enhanced the transdermal permeation of IBU sodium over the first 6 h (~ 5.11 mg) when compared to MN alone (~ 1.63 mg) (*p* < 0.05). In contrast, heat-assisted technology showed almost no promoting effect on transdermal delivery. Furthermore, IBU sodium-containing rapidly dissolving lyophilised and effervescent reservoirs, classified as chemical modification methods, were prepared. Both strategies achieved rapid and effective ex vivo IBU sodium permeation, equating to ~ 78% (30.66 mg) and ~ 71% (28.43 mg) from lyophilised and effervescent reservoirs, respectively. Moreover, in vivo pharmacokinetic studies showed that the IBU sodium plasma concentration within lyophilised and effervescent groups reached a maximum concentration (C_max_) at 4 h (~ 282.15 µg/mL) and 6 h (~ 140.81 µg/mL), respectively. These strategies not only provided rapid achievement of therapeutic levels (10–15 µg/ml), but also resulted in sustained release of IBU sodium for at least 48 h, which could effectively reduce the frequency of administration, thereby improving patient compliance and reducing side effects of IBU sodium.

**Graphical Abstract:**

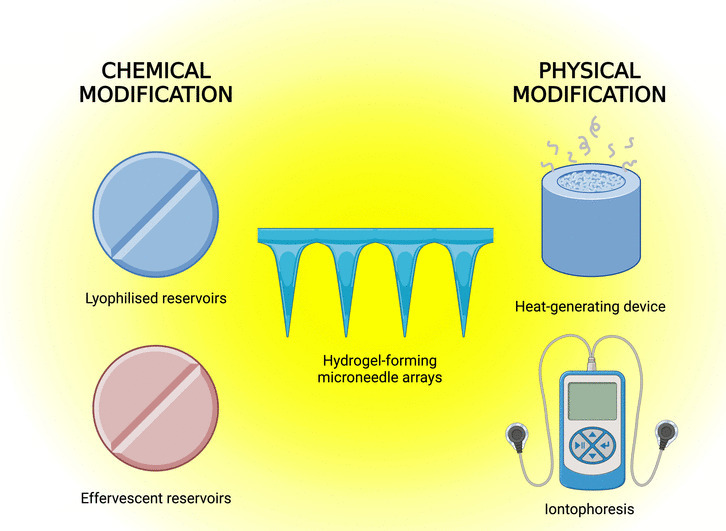

**Supplementary Information:**

The online version contains supplementary material available at 10.1007/s13346-024-01737-0.

## Introduction

**Fig. 1:**
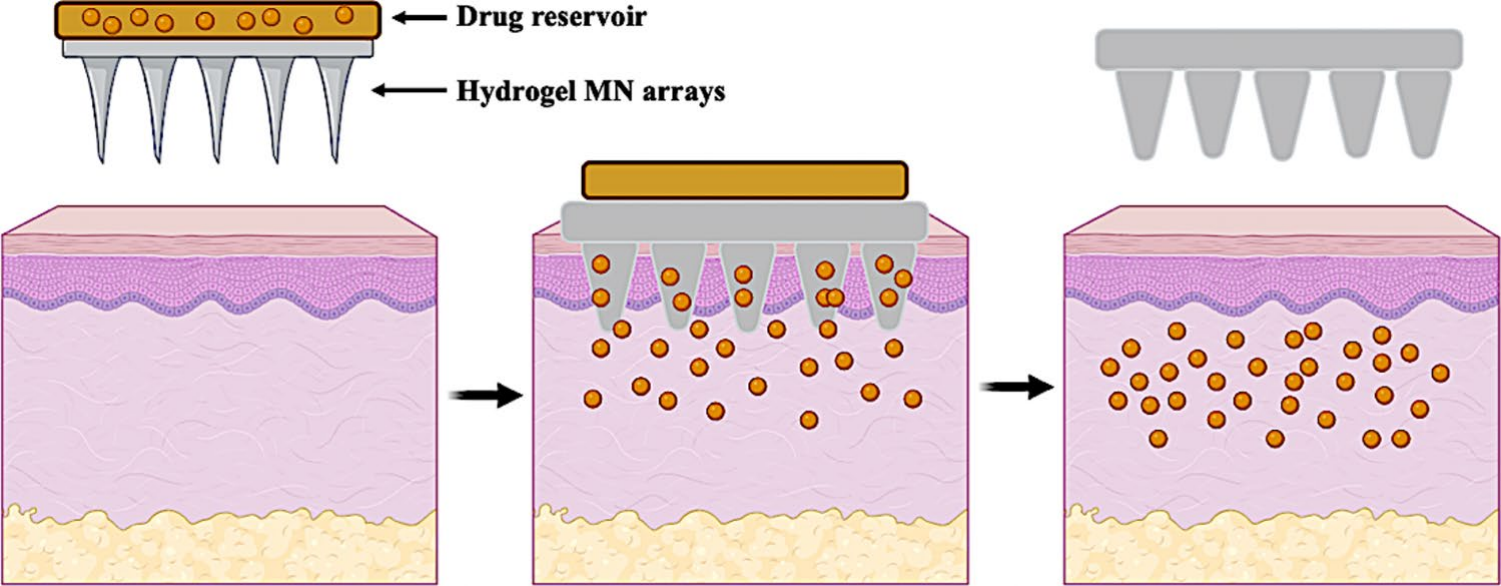
Schematic representation of hydrogel-forming MNs. When applied to the skin, these MN arrays are designed to rapidly absorb interstitial fluid from the skin and swell, resulting in the formation of continuous hydrogel microchannels that allow therapeutic drugs to diffuse from the top reservoir into the dermal microcirculation. Reproduced with permission [5]

Transdermal drug delivery (TDD), as an alternative to oral and parenteral administration, can circumvent associated issues such as first-pass effect and needle phobia. However, due to the skin barrier effect, only drugs with specific properties, such as low molecular weight (MW) (< 500 Da) and balanced lipophilicity (LogP 1–3), can passively diffuse through the *stratum corneum* (*SC*), thereby limiting the application of TDD [1] Microneedle (MN) arrays offer an option to overcome this obstacle, as they exhibit the ability to bypass the *SC* and allow drugs to diffuse into the deep skin layers [2]. Hydrogel-forming MN arrays represent one of the five designs within MN technology and permit the delivery of high doses of drug molecules across the skin. Within this MN design, the active substances are loaded into a separate drug reservoir meaning that the loading capacity is not limited to the MN needles themselves [3, 4]. The structure of hydrogel-forming MNs and their mechanism are schematically in Fig. 1. Previous studies have shown that a wide range of drug reservoirs can be integrated into hydrogel-forming MN arrays for different transdermal delivery purposes [3–11]. However, when compared to conventional needles and syringes, hydrogel-forming MN technology typically exhibits a lag phase, resulting in a slower rate of drug delivery. Therefore, the present work highlights different physical and chemical modification methods to drug reservoirs in combination with hydrogel-forming MN arrays, to further improve the amount and rate of delivery of a small molecule water-soluble model drug, ibuprofen (IBU) sodium, to enable rapid TDD.

When considering the limited amount of moisture available from the swollen hydrogel-forming MN arrays, the concept of rapidly dissolving reservoirs is a key feature [12]. In this work, two different types of IBU sodium-containing reservoirs were considered, namely lyophilised and effervescent reservoirs. Both systems are expected to release their cargo immediately through swollen hydrogel-forming MN arrays after skin application to achieve rapid TDD. Lyophilisation, known to improve dissolution rates and solubility properties of drug substances [13–15], generates porous, hygroscopic products during the freeze-drying process. Moreover, the organic acids and carbonates contained in effervescent tablets react immediately upon contact with water, producing a large amount of carbon dioxide (CO_2_) which results in the disintegration of tablets. Rapid in vitro disintegration and dissolution are the most prominent feature of clinical studies on effervescent tablets. In addition, effervescent compounds that produce gases may achieve deep drug penetration. This was shown by Ke et al.. (2012), in which a MN system incorporating NaHCO_3_ and rapidly formed CO_2_ bubbles was designed [16]. This study indicated that bubble generation can actively push the drug deep into the skin, highlighting the broad prospects of TDD.

In addition to chemical modification methods of drug reservoirs, physical permeation enhancement techniques, such as iontophoresis (ITP) combined with MNs, have shown promising results. An in vivo study on the transdermal permeation of leuprorelin acetate using ITP combined with MNs found that the drug plasma levels detected were three times higher than with MNs alone, as well as facilitating faster drug penetration [17]. It has also been proposed that combining MN and ITP can achieve rapid delivery of hormones and vaccines, in addition to precise control of the delivery of conventional drug substances [18, 19].

In pain management, it has often been found that applying localised heat prior to the topical application of therapeutic drugs (such as fentanyl) significantly improves drug absorption [20, 21]. This suggests that heat is thought to enhance transdermal drug absorption by increasing blood perfusion and affecting drug delivery parameters across the skin [22, 23]. Otto and de Villiers (2013) reported that heat can increase the diffusion coefficients of drug molecules, leading to higher diffusivity and increased drug release [22]. Several heating devices have been developed for this purpose. Nuvo Research Inc. created a controlled heat-assisted drug delivery (CHADD™) technology that has been approved for several products, such as Synera^®^, used for transdermal delivery of lidocaine and tetracaine. It has been reported that the CHADD patch generates heat through iron powder oxidation, which can rapidly increase serum fentanyl concentration and shorten the time to reach therapeutic levels [20]. Consequently, in this present work, we also investigated the potential of both ITP technology, and a heat-assisted drug delivery technology based on iron powder oxidation to accelerate IBU sodium TDD when combined with hydrogel-forming MN arrays.

In summary, hydrogel-forming MNs allow for effective TDD with higher doses through the use of a separate drug reservoir [3, 4]. However, due to their potentially slower delivery rate compared to syringes, this study explored various enhancement strategies in combination with hydrogel MNs. Chemical modification methods, such as lyophilised and effervescent reservoirs, were designed to accelerate drug release upon hydration of the MN arrays. Additionally, physical techniques, including ITP and a heat-assisted delivery system, were also investigated. These combined approaches aim to facilitate rapid and efficient TDD of ibuprofen sodium.

## Materials and methods

### Materials

Gantrez^®^ S-97, a copolymer of methylvinylether and maleic acid (PMVE/MA), MW 1.5 × 10^6^ Da and Plasdone^®^ K-29/32, poly(vinyl pyrrolidone) (PVP) MW 5.8 × 10^4^ Da, were gifts from Ashland Specialties UK Ltd, Kidderminster, Worcestershire. Gelatin, sold under product brand name Cryogel^®^ SG/3 was provided from PB Gelatins GmbH, Nienburg/Weser, Germany. Mannitol, sold under product brand name Pearlitol^®^ 50 C, was purchased from Roquette, (Lestrem, France). All other chemicals and materials were of analytical grade and purchased from Sigma-Aldrich (Dorset, UK) or Fisher Scientific (Loughborough, UK).

### Fabrication of hydrogel films

Stock solutions of 40% w/w Gantrez^®^ S-97, 25% w/w PVA 85–124 kDa and 40% w/w PVP K29/32 were prepared with deionised water. The different formulations of Gantrez^®^ S-97 and PVA-based hydrogel films are listed in Table 1 and were prepared as previously described [8, 12, 24]. All aqueous blends were centrifuged (Eppendorf^®^ 5804 series centrifuge, Fisher Scientific, Loughborough, UK) at 3,500 rpm for 15 min to remove the air bubbles. Approximately 0.5 g of each hydrogel formulation was carefully poured into a flat mould (1 cm^2^ square). All moulds were then centrifuged at 3,500 rpm for 15 min and placed on a flat surface. After 48 h of room temperature drying, the hydrogel films were removed from the moulds. Afterwards, the Gantrez^®^ S-97-based films (H1 and H2) were crosslinked for 24 h at 80 °C. In the case of PVA-based hydrogel films, there are two formulations with different crosslinking times, 130 °C for 40 min (H3) and 130 °C for 3 h (H4) to induce the esterification reaction [25–27]. During this process, the hydroxyl groups in PVA and PEG 10,000 reacted with the carboxyl groups in citric acid and Gantrez^®^ S-97, respectively, forming ester bonds between polymer chains and resulting in a three-dimensional crosslinked network [25–27].

**Table 1:** Hydrogel formulations and the conditions of crosslinking

Hydrogel formulation	Composition (%w/w)	Temperature (°C)	Crosslinking time
H1	20% Gantrez^®^ S-97, 7.5% PEG 10,000, 3% Na_2_CO_3_	80	24 h
H2	20% Gantrez^®^ S-97, 7.5% PEG 10,000	80	24 h
H3	15% w/w PVA, 10% w/w PVP, 1.5% w/w citric acid	130	40 min
H4	15% w/w PVA, 10% w/w PVP, 1.5% w/w citric acid	130	3 h

### Swelling studies of hydrogel films

Swelling studies were performed as previously published [12, 28]. Initially, 1 cm^2^ hydrogel films were weighed and recorded as m_0_ in the dry state (t = 0), followed by immersion in PBS (pH 7.4) with a known volume (20 mL) over 24 h at room temperature. Films were removed periodically, and excess surface PBS (pH 7.4) was blotted using filter paper. The weight of each film was then remeasured and recorded as m_t_. The swelling percentage was obtained using Eq. 1.

1 $$\:\:\:Swelling\:\left(\%\right)=\:\frac{{(m}_t\:-\:m_0)}{m_0}\:\times\:\:100\%$$

The percentage of equilibrium water content (EWC) was also obtained in this study. The hydrogel samples were weighed and recorded as m_s_ in the equilibrium state where there is no further change in hydrogel mass. The % EWC was calculated using Eq. 2.

2 $$\:\:EWC\:\left(\%\right)=\:\frac{{(m}_s\:-\:m_0)}{m_s}\:\times\:\:100\%$$

### Solute diffusion studies

Solute diffusion studies were performed to assess the diffusion capacity of IBU sodium across swollen membranes of different hydrogel formulations using side-by-side type diffusion cells (Permergear, Hellertown PA, USA), with the aim of identifying the most promising formulation for the fabrication of hydrogel-forming MN arrays. In this experiment, after an overnight pre-equilibration period in PBS (pH 7.4), each hydrogel film was cut with scalpels into a circular shape of approximately 9 mm^2^ in diameter. Following that, each film was clamped between two half-cells and coated using Parafilm^®^ M to prevent evaporation and leakage. Afterwards, the donor compartment was filled with 3 mL IBU sodium solution of 1 mg/mL in PBS (pH 7.4), and the receptor compartment was filled with 3 mL of PBS (pH 7.4). At each specified time point (1, 2, 3, 4, 6, 24 h), 3 mL of the samples were withdrawn from the receptor compartment and replaced with the same volume of fresh pre-heated PBS (pH 7.4). The removed aliquots of IBU sodium were subsequently analysed using the validated high-performance liquid chromatography (HPLC) method detailed in Sect. 2.16.

### Fabrication of hydrogel-forming MN arrays

Hydrogel-forming MNs were fabricated from the aqueous blends of four hydrogel formulations (H1-H4) previously mentioned in Sect. 2.2. Approximately 0.5 g of each hydrogel formulation was dispensed into polydimethylsiloxane moulds consisting of 121 (11 × 11) conical needles with base width of 300 μm, height of 600 μm and interspacing of 300 μm. The moulds were then centrifuged at 3,500 rpm for 15 min to remove surface bubbles and ensure homogeneous distribution of the gels within the micropores of the moulds. Following that, they were left to dry at room temperature. After 48 h, the MN arrays were carefully removed from the moulds and then crosslinked at 80 °C for 24 h (H1 and H2), at 130 °C for 40 min (H3) and 3 h (H4) as indicated in Table 1. Lastly, a heated scalpel was used to carefully remove the sidewalls of the MN arrays formed during the moulding process.

### Mechanical characterisation and insertion studies

Mechanical characterisation of MN arrays was assessed using the TA.XT2 Texture Analyser (Stable Microsystems, Haslmere, UK) in compression mode [5, 29, 30]. The MN arrays were visualised before and after testing using the Leica EZ4 D digital microscope (Leica Microsystems, Milton Keynes, UK) and the height of a minimum of 5 needles from each side of the MNs was measured and recorded. The percentage of the height reduction was determined using Eq. 3 below.

3 $$\:\:Height\:reduction\:\left(\%\right)=\:\frac{H_0\:-\:H_1}{H_0}\:\times\:\:100\%$$

where H_0_ is the height before testing and H_1_ is the height after testing.

Insertion studies were also conducted using the TA.XT2 Texture Analyser. Briefly, eight layers of Parafilm^®^ M with a thickness of approximately 1 mm, as a verified skin model [31, 32], were placed on the stainless-steel baseplate. After attaching the MN arrays to the probe, the probe was lowered until the 32 N force was reached and then held for 30 s, after which the MN arrays were removed. Subsequently, the layers of Parafilm^®^ M were separated from each other and the number of holes in each layer counted under a light microscope. The insertion percentage was obtained using the following Eq. 4.

4 $$\:\:Insertion\:\left(\%\right)=\:\frac{number\:of\:holes\:in\:each\:layer}{numbers\:of\:needles\:in\:an\:MN\:array}\:\times\:\:100\%$$

Moreover, optical coherence tomography (OCT) using an EX1301 VivoSight^®^ OCT microscope (Michelson Diagnostics Ltd, Kent, UK) was used to evaluate the insertion ability of MN arrays into full-thickness neonatal porcine skin, which is used as an alternative for human skin [31, 32]. The MN arrays were inserted into the skin through manual thumb pressure for 30 s and then visualised using OCT. An imaging software, Image J^®^ (National Institutes of Health, Bethesda MD, USA), was used to determine the height of the inserted needles.

### Preparation of lyophilised reservoirs

Lyophilised reservoirs are tablets which have the characteristics of rapid disintegration due to their porous structure [13–15]. Various formulations of gelatin and mannitol were evaluated for their potential use in lyophilised reservoirs loaded with IBU sodium, as presented in Table 2. A powder blend made from the mixture of IBU sodium, mannitol and gelatin was homogenised using a mortar and then transferred to a mixing container. After that, an appropriate amount of deionised water was added to the dry powder mixture to achieve the required total mass of the reservoir. The aqueous mixture was homogenised using a SpeedMixer™ DAC 150.1 FVZ-K (GermanEngineering, Hauschild & Co. KG, Hamm, Germany) at 3,000 rpm for 5 min and then sonicated at ~ 37 °C for 15 min. Approximately 250 mg of the aqueous mixture was cast into open-ended cylindrical moulds with a diameter of 8 mm and a depth of 4 mm. The cast mixtures were then pre-frozen at -80 °C for 3 h to prepare samples for the lyophilisation process. Afterwards, these samples were lyophilised using a Virtis Advantage Bench Top Freeze Drier System (SP Scientific, Warminster, PA, USA) for 24 h.

**Table 2:** Composition of IBU sodium lyophilised reservoirs (LF1-LF6) produced using different combinations of mannitol, gelatin and IBU sodium,

Formulation	Composition (%w/w)			
	Mannitol	Gelatin	IBU sodium	Deionised water
LF1	3	10	40	47
LF2	10	5	40	45
LF3	10	5	45	40
LF4	5	10	40	45
LF5	3	5	40	52
LF6	3	10	45	42

### Preparation of effervescent reservoirs

Effervescent reservoirs are tablets which are designed to dissolve in water and release CO_2_. The direct compression method was chosen to prepare effervescent reservoirs using a manual hydraulic press (Specac^®^ Atlas, Specac Ltd, Kent, UK). The different formulations are listed in Table 3. Prior to compression, the powder mixture of IBU sodium, acid sources, and alkali metal carbonates in different proportions of different formulations were weighed, and then ground in a mortar for 3 min to mix evenly and form a fine powder. Approximately 100 mg of powder mixture from each formulation were accurately weighed and then manually loaded into the tablet die. Using the manual hydraulic press, 5 t (metric ton) of pressure was applied for 30 s to each effervescent reservoir formulation. Following removal from the die, the fully formed tablets were visually examined using a light microscope.

**Table 3:** Composition of IBU sodium effervescent reservoirs (EF1-EF8) produced using different disintegrants and IBU sodium loadings

Formulation	Composition (%w/w)				
	Citric acid	Tartaric acid	Na_2_CO_3_	NaHCO_3_	IBU sodium
EF1	35	−	−	35	30
EF2	−	35	−	35	30
EF3	−	35	35	−	30
EF4	35	−	35	−	30
EF5	7.5	2.5	15	35	40
EF6	5	5	25	25	40
EF7	7.5	2.5	35	15	40
EF8	2.5	7.5	35	15	40

### Preparation of IBU sodium-loaded polymeric films

The IBU sodium-loaded films used as thermal/electro-responsive reservoirs for ITP/heating-mediated delivery were prepared using a casting method from aqueous blends containing IBU sodium, different polymers and deionised water in different proportions (Table 4). First, the aqueous mixture was placed in a mixing container and homogenised using a SpeedMixer™ DAC 150.1 FVZ-K at 3,000 rpm for 5 min, and then sonicated at ~ 37 °C for 5 min to remove the bubbles. Afterwards, 7.5 g of the aqueous blends containing IBU sodium was slowly cast into a pre-levelled glass rectangular mould (internal dimensions of 30 mm × 50 mm), which was lined with a release liner to facilitate removal of the film. The IBU sodium-loaded film was subsequently removed from the glass rectangular mould after drying at room temperature (25 °C) for 48 h.

**Table 4:** Different formulations investigated (F1-F14) to produce IBU sodium-loaded polymeric films

	Formulation composition (% w/w)								
	IBU sodium	Glycerol	PVA 85–124 kDa	PVP K29/32	PVA 9–10 kDa	PVP k-90	PVA 31–50 kDa	GantrezS-97	PEG 10,000
F1	5	−	15	15	−	−	−	−	−
F2	5	−	−	15	15	−	−	−	−
F3	10	−	−	15	15	−	−	−	−
F4	10	15	−	−	−	15	−	−	−
F5	15	15	−	−	−	15	−	−	−
F6	5	10	−	−	−	−	15	−	−
F7	7.5	10	−	−	−	−	15	−	−
F8	5	15	−	−	−	−	15	−	−
F9	7.5	15	−	−	−	−	15	−	−
F10	10	15	−	−	−	−	15	−	−
F11	2.5	−	−	−	−	−	−	20	10
F12	5	−	−	−	−	−	−	20	10
F13	7.5	−	−	−	−	−	−	20	10
F14	10	−	−	−	−	−	−	20	10

### Reservoir characterisation

The appearance of IBU sodium-loaded lyophilised and effervescent reservoirs were visualised using a Leica EZ4 D stereomicroscope. Hardness of all reservoirs was determined using the Copley Hardness Tester (Dr. Schleuniger Pharmatron, Copley Scientific, Nottingham, UK). The weight variation test of reservoirs was also conducted, in which twenty tablets were selected at random, weighed individually and the average weight was calculated. The percentage friability of reservoirs was determined using the friability tester (Copley Scientific, Nottingham, UK). Mechanical analysis of IBU sodium-loaded films was performed in terms of tensile strength and percentage elongation at break using a TA.XT2 Texture Analyser in tensile mode. The dissolution time for each reservoir determined visually [3, 4]. In brief, each reservoir was placed in a glass vial containing 25 mL of PBS (pH 7.4), stirred with a magnetic stirring bar (6 mm × 25 mm) at 600 rpm, with the temperature maintained at 37 °C throughout the experiment. The time required for complete dissolution of each reservoir was recorded as the dissolution time [8]. For recovery percentages, the reservoirs were placed in glass vials with 25 mL of PBS and kept in the shaker incubator at 37 °C overnight to ensure complete dissolution. Afterwards, the dissolution medium was sampled, with samples diluted, filtered using 0.22 μm Millex^®^-GS syringe filters and quantified using the validated in vitro HPLC method. Furthermore, the pure IBU sodium, pure excipients and IBU sodium-containing reservoirs were subjected to differential scanning calorimetry (DSC) and attenuated total reflectance-fourier transform infrared (ATR-FTIR) analysis using the DSC Q100 (TA Instruments, Elstree, Hertfordshire, UK) and an ATR-FTIR spectrometer (Accutrac FT/IR-4100™, Perkin Elmer, USA), respectively. Among them, DSC analysis was performed under nitrogen flow at a scanning rate of 10 °C/min, ranging from 25 °C to 300 °C. And ATR-FTIR spectrum was obtained and recorded in the range of 4000 –400 cm-1 at room temperature with a resolution of 4.0 cm-1. The resulting spectra were the average of 32 scans.

### Heating properties of heat-generating powder mixtures

The heating properties of the powder mixtures were evaluated from two parameters, namely the maximum heating temperature and duration of temperature above 37 °C. The temperature of the heat-generating chemical powder mixture from the different formulations was measured using an RS40 Wired Digital Thermometer (RS Components Ltd. Birchington Road, Corby, Northants, UK). Initially, the chemical powder mixture was placed in a glass vial and mixed using a Vortex™ (Fisons Scientific Equipment, Loughborough, Leicestershire, UK) for 1 min. Afterwards, an appropriate amount of water was added to the glass vial. The vial was then vortexed for 30 s and sealed using Parafilm^®^ M and aluminum foil, with a hole approximately 5 mm diameter for temperature measurements. The probe tip of the thermometer was passed through the hole and placed in the middle region of the powder mixture to measure the real-time temperature. The heat was generated after approximately 1 min. The highest temperature of the heat-generating powder mixture was recorded. Meanwhile, the duration of the powder mixture temperature above 37 °C was recorded as the heating duration.

### Preparation of heat-generating devices

**Fig. 2:**
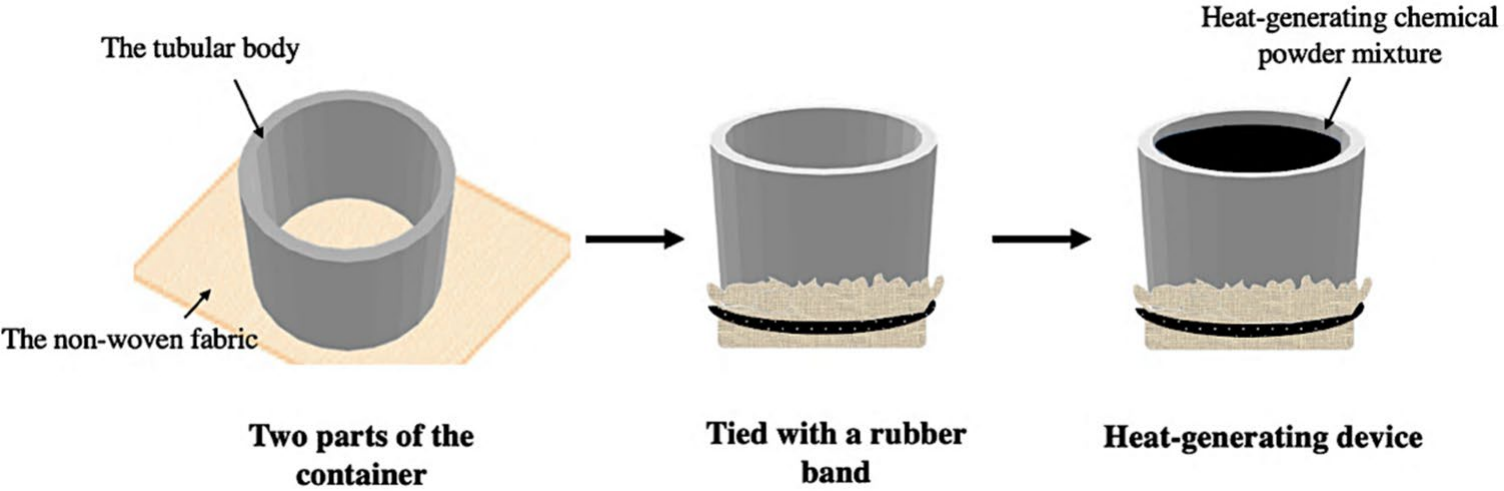
Schematic illustrations for the preparation of heat-generating device

The heat-generating device included a container and the heat-generating chemical powder mixture. The container used to hold the heat-generating powder mixture was designed and fabricated. This was divided into two parts, a tubular body and a non-woven bottom, as displayed in Fig. 2. Initially, the tubular body part was designed using Tinkercad^®^ software and printed using an UltimakerCura 4.4 three-dimensional (3D) printer. A poly(propylene) filament was chosen as the printing material and connected to the printer. The dimensions of the tubular body were 16.5 mm in height and 21.5 mm in diameter. A piece of non-woven fabric was then connected to one end of the tubular body by a rubber band, which was used as the bottom. Finally, the heat-generating powder mixture was directly placed into the container to complete the heat-generating device.

### Ex vivo permeation studies

**Fig. 3:**
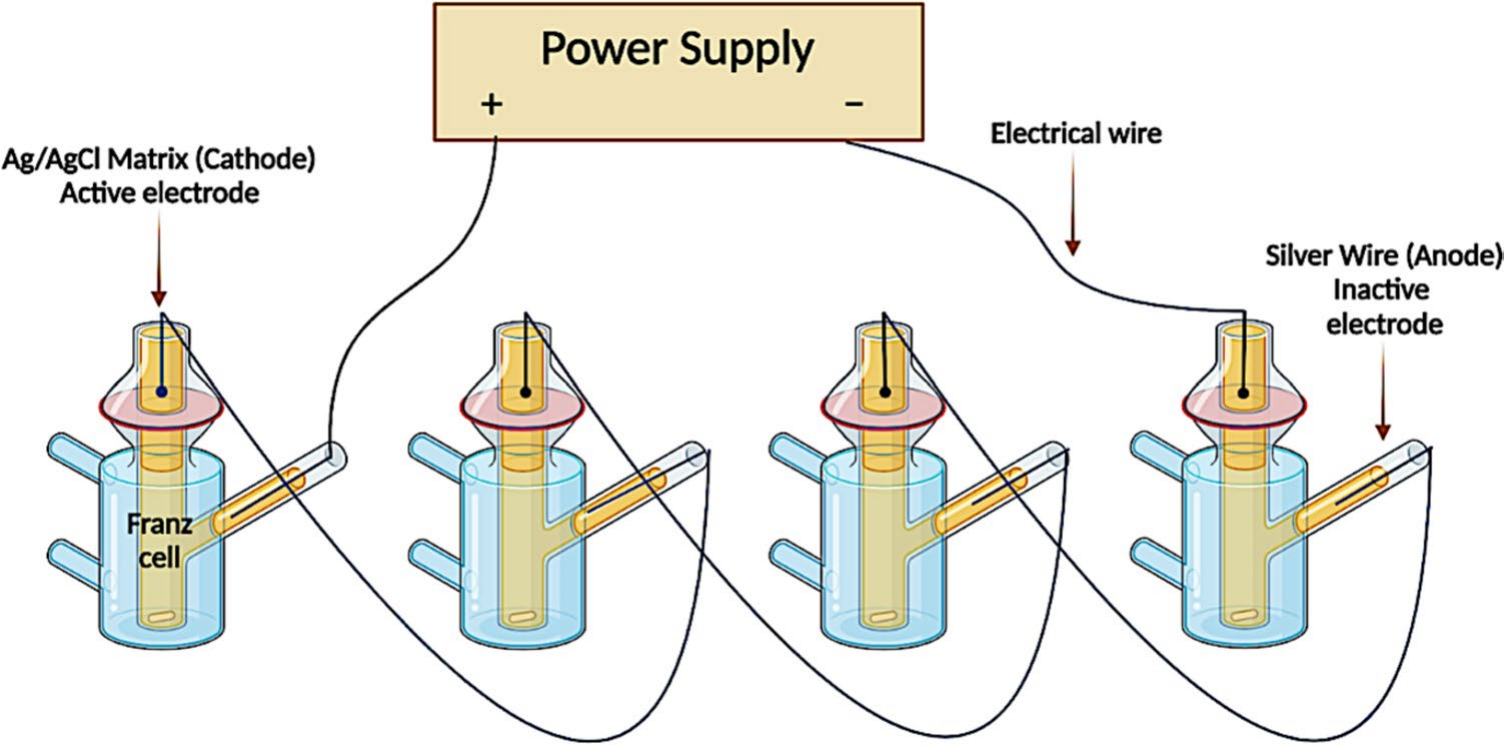
Schematic illustration of the experimental setup employed during ex vivo iontophoretic investigations

For ex vivo permeation studies, modified Franz diffusion cells were used to evaluate the permeation of IBU sodium across dermatomed (350 μm) neonatal porcine skin using different hydrogel-forming MN array setups. Dermatomed skin was obtained from the dorsal area of stillborn piglets within 24 h after birth using an electric dermatome trimmer (Integra Padgett^®^ model B, Integra Life Sciences Corporation, Ratingen, Germany) and stored at -20 °C until further required. Prior to the experiment, the skin was defrosted and pre-equilibrated in PBS (pH 7.4) for 30 min and then attached carefully to the donor compartment of the Franz diffusion cells using cyanoacrylate glue, with the epidermis side facing up. Subsequently, the hydrogel-forming MN arrays, prepared from an aqueous blend of 15% w/w PVA 85–120 kDa, 10% w/w PVP K29/32 and 1.5% w/w citric acid, were inserted into the skin using thumb pressure for 30 s. Then, a 20 µL aliquot of water was added to the top of the MN arrays prior to placement of the lyophilised, effervescent or polymeric IBU sodium-loaded reservoirs (thermal/electro-responsive drug-containing films), which promoted the adhesion of the reservoirs to the baseplate of the MN arrays. Afterwards, a stainless steel cylinder (11 mm in diameter and 11.5 g in mass) was placed on top of the reservoir and MN to keep the device stationary and prevent MN expulsion upon its swelling over time. The receiver compartment was filled with 12 mL of PBS (pH 7.4) release media, which was preheated and maintained at 37 °C during the whole experiment. The donor compartment was then clamped to the top of the receiver compartment. To prevent the evaporation of the release media, the donor compartment and the sampling arm of the receiver compartment were sealed with Parafilm^®^ M. In control cells, only reservoirs were placed on the top of the skin. At pre-determined time intervals (1, 2, 4, 6, 8, 24 h), 200 µL of release media was withdrawn from each side arm of the receiver compartment and replaced with 200 µL of fresh preheated PBS. All the samples were diluted appropriately, if required, then centrifuged at 14,800 rpm for 15 min and analysed using the validated HPLC method. For the application of an electrical current, the silver wire was used as an anode, with a silver/silver chloride electrode acting as the cathode. The cathode was placed directly on top of the integrated hydrogel-forming MNs which consist of a drug-loaded adhesive reservoir and a drug-free hydrogel MN array [5] in the donor compartment, whilst the anode was placed into the release medium in the receiver compartment *via* the side arm of the Franz cell. Two different current densities of 0.1 and 0.5 mA/cm^2^ were applied for a period of 6 h using a Phoresor II Auto power supply (Iomed, Lake City, FL, USA). Figure 3 displays an illustration of the experimental setup employed during the iontophoretic investigations. Regarding the heat-assisted combined delivery, after applying the hydrogel-forming MN system into dermatomed neonatal porcine skin within the Franz cell donor compartment, the heat-generating device was directly placed on top of the integrated hydrogel-forming MN. Then, the donor was sealed with Parafilm^®^ M and aluminum foil to maintain heat.

### In vivo delivery of IBU sodium

Approval for the animal studies was obtained from the Committee of the Biological Services Unit, Queen’s University Belfast under Project Licence PPL 2903, and Personal Licences PIL 2127 and PIL 2059. All in vivo experiments throughout this study were performed according to the policies of the Federation of European Laboratory Animal Science Associations and the European Convention for the Protection of Vertebrate Animals used for Experimental and other Scientific Purposes, with strict adherence to the principles of the 3R’s (replacement, reduction, and refinement). All 18 female Sprague-Dawley rats, aged 8–10 weeks and with an average weight of 255 g, were subjected to a one-week acclimatisation period prior to commencing the experimental procedures. This study consisted of three groups, each containing 6 rats. Each rat in the first group received an oral gavage of Nurofen^®^ oral IBU suspension (20 mg/mL) at a dose of 60 mg/kg. The second group received four lyophilised reservoirs combined with hydrogel-forming MNs for each rat at a dose of 160 mg. The last group received four effervescent reservoirs in combination with hydrogel MNs, at a dose equivalent to 160 mg as well. After removing dorsal hair, hydrogel-forming MNs were then inserted into a pinched section of dorsal skin on rats from both Group 2 and 3 using firm finger pressure. Prior to placing the IBU sodium-containing reservoirs (lyophilised and effervescent reservoirs), which were secured inside the Microfoam™ adhesive frames, a 20 µL aliquot of water was added to the centre of the MN arrays. This promoted the adhesion of the reservoirs to the baseplate surface. Afterwards, Tegaderm™ film was placed on top of the Microfoam™ adhesive frames, followed by wrapping kinesiology tape around the back of the rats to secure the entire system in place for 5 days. Blood samples were then obtained from the tail veins of the rats into 1.5 mL heparinised Eppendorf^®^ tubes at pre-determined time points over 5 days (1, 2, 4, 6, 24, 48, 72, and 120 h). Upon completion of the animal study on Day 5, the drug reservoirs and hydrogel-forming MNs were removed from the rats in Group 2 and 3, followed by euthanasia of all animals using carbon dioxide asphyxiation.

### IBU sodium extraction procedure from rat plasma

The whole blood was drawn from the rats and immediately centrifuged at 1,500 rpm for 15 min at 4 °C. The supernatant plasma was collected and stored at -20 °C until further extraction and analysis. For each drug extraction, 0.3 mL of acetonitrile was added to the micro tube containing 100 µL of plasma, followed by vortex-mixing for 10 min and centrifuging at 14,000 g for 10 min at 4 °C. Furthermore, the supernatant was separated and transferred into a disposable glass culture tube. The extract was subsequently subjected to a drying process for 50 min at 37 °C under a nitrogen stream using the Zymark TurboVap^®^ LV Evaporator Workstation. Thereafter, the residue obtained was reconstituted in a 1.5 mL micro tube with 100 µL of PBS and sonicated for 30 s to ensure complete dissolution. This was followed by centrifugation at 14,000 g for 10 min at 25 °C. Finally, the supernatant was analysed using validated method of HPLC.

### Instrumentation and chromatographic conditions

The detection and quantification of IBU sodium was conducted using an Agilent HPLC 1200 series system. A Phenomenex^®^ Luna C18 (ODS1) column (150 mm length, 4.6 mm width and 5 μm pore size) was used as the stationary phase for isocratic separations of IBU sodium. Table 5 outlines all the chromatographic conditions that were used to achieve effective chromatographic separation. In addition, the Agilent ChemStation^®^ Software B.02.01 was used to analyse the obtained chromatograms.

**Table 5:** The chromatographic conditions used for effective chromatographic separation of IBU sodium

Samples	IBU sodium dissolved in PBS (pH 7.4)
Mobile phase	Acetonitrile and 0.1% phosphate acid at pH 2.24 (70%:30%)
Injection volume	50 µL
Run time	9 min
UV detection	223 nm
Flow rate	1 mL/min
Column temperature	25 °C

### Pharmacokinetic analysis

Non-compartmental pharmacokinetic analysis (PK analysis) of IBU sodium plasma concentrations was performed using PK Solver, an add-in tool for Microsoft^®^ Excel (Microsoft Corporation, Redmond, WA, USA). The maximum plasma concentration (C_max_) of IBU sodium and the corresponding time of occurrence (T_max_) were directly determined by inspecting the raw data. Moreover, the area under the curve from time zero to the last time point (AUC_0−t_) was determined using the linear trapezoidal method.

### Statistical analysis

Version 9.0 GraphPad Prism^®^ (GraphPad Software, San Diego, California, ) was used for statistical analysis, including the calculation of means and standard deviation. An unpaired t-test was performed to analyse two groups, one-way analysis of variance (ANOVA) and two-way ANOVA with Tukey’s post hoc were used to analyse the differences among different groups (≥ 3 groups). Statistical significance was shown by *p* < 0.05 in each instance.

## Results and discussion

### Swelling studies of hydrogel films

**Fig. 4:**
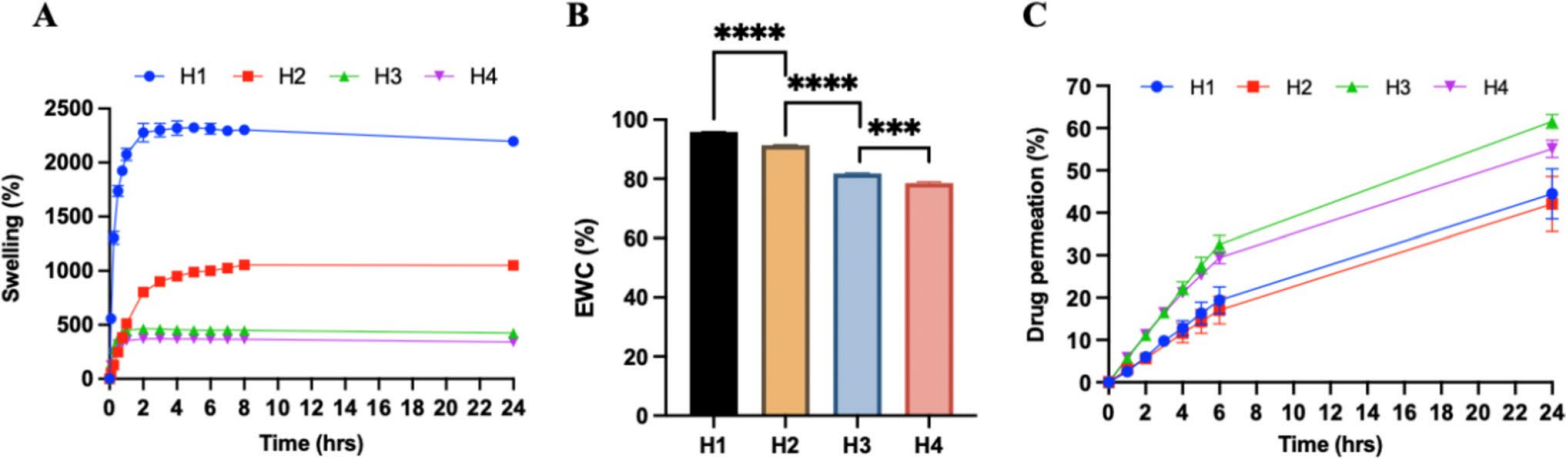
(**A**) Swelling profiles of crosslinked hydrogel films prepared from different formulations (H1-H4) over 24 h (means ± SD., *n* = 3). (**B**) % EWC of hydrogel films for H1-H4 (means + SD., *n* = 3). (***p < 0.001, ****p < 0.0001). (**C**) Percentage permeation of IBU sodium across pre-swollen hydrogel films (means ± SD., *n* = 3)

The swelling properties of hydrogels have an impact on their mechanical properties and the capability of drug diffusion [8, 33]. Figure 4A displays the swelling profiles of four different hydrogel formulations (H1-H4) over 24 h. It can be demonstrated that Gantrez^®^ S-97-based hydrogel films of H1 and H2 exhibited a significant superiority in swelling capacity compared to PVA-based hydrogel formulations (H3 and H4) (*p* < 0.05). The crosslinking ratio is a critical factor influencing the swelling properties of hydrogels [25, 34], which refers to the molar ratio between crosslinker and polymer repeat units [34]. The higher the degree of crosslinking, the lower the swelling degree of hydrogels [35]. In this case, the higher crosslinking ratio observed in PVA-based formulations in comparison with Gantrez^®^ S-97-based formulations resulted in a more compact crosslinked network structure, which restricted the movement of the polymer chain, thus leading to a lower swelling degree [8, 36].

In addition, when comparing the two formulations that both contained Gantrez^®^ S-97 as the polymeric backbone, it was observed that H1 had a significantly higher percentage swelling than H2 (*p* < 0.05). At equilibrium, the percentage swelling was 2303% and 1055% for H1 and H2, respectively. This is attributed to the addition of a modifying agent, Na_2_CO_3_, in H1, which was responsible for the reduction in the degree of esterification, thereby forming a more porous network with an increased swelling capacity in comparison with H2. Meanwhile, osmotic pressure is the driving force for water to enter the polymer network of hydrogels, while the presence of dissociated sodium carboxylate groups in the hydrogel network of H1 enhanced the osmotic pressure, thus increasing its swelling potential [37, 38]. The results are consistent with the previously published report [12].

Furthermore, comparing H3 and H4, which had the same composition (15% w/w PVA, 10% w/w PVP and 1.5% w/w citric acid) but different crosslinking time, the percentage swelling of H3 (crosslinked for 40 min) was 448% at equilibrium, which was significantly higher than 366% for H4 (crosslinked for 3 h) (*p* < 0.05). This may be explained by the formation of denser ester bonds between citric acid and PVA during extended crosslinking time. It has been observed in previous studies that prolonging the crosslinking time can improve the degree of crosslinking, thereby reducing swelling degree [39, 40].

The EWC percentage was determined as a measure of the water absorption capacity of the hydrogel films from different formulations. The results are presented in Fig. 4B. Similar to the percentage swelling results, the significantly higher EWC percentage was observed in H1 and H2, both containing Gantrez^®^ S-97, compared to H3 and H4 (PVA-based formulation) (*p* < 0.05). Moreover, the EWC percentage was significantly different between H1 and H2, as well as between H3 and H4 (*p* < 0.05). As previously mentioned, this is due to the addition of Na_2_CO_3_ in H1 and the reduced heating time in H3, forming the lowly crosslinked systems, allowing for greater water absorption into the network structure of the hydrogels [12, 41].

### Solute diffusion studies

This study evaluated the permeation of IBU sodium across the swollen hydrogel membranes of different formulations at equilibrium using side-by-side diffusion cells. The permeation profiles of H1-H4 are presented in Fig. 4C. Compared to Gantrez^®^ S-97-based films, the permeation percentage of IBU sodium across the PVA-based swollen films was significantly higher (*p* < 0.05). After 24 h, ~ 61% (1.85 mg) and ~ 55% (1.64 mg) of IBU sodium had permeated across the swollen PVA-based films of H3 and H4, respectively, whereas in the case of H1 and H2 (Gantrez^®^ S-97-based formulations), only 45% and 42% of IBU sodium were detected in the receptor compartment, corresponding to approximately 1.34 mg and 1.26 mg, respectively. This could possibly be attributed to the fact that Gantrez^®^ S-97, which has a much higher MW than PVA, results in an increased molar ratio of the hydroxyl groups of the polymer to the carboxyl groups of IBU sodium, thus enhancing hydrogen bonding interaction between the IBU sodium and Gantrez^®^ S-97 within the 3D hydrogel network [42]. Consequently, solute entrapment occurred in the Gantrez^®^ S-97-based gel system (H1 and H2), thus reducing the cumulative amount of IBU sodium permeated across the films [41, 43–45]. This also explains why Gantrez^®^ S-97-based films have a higher swelling degree but a lower solute diffusion capacity than PVA-based films. The results of this study are in agreement with the findings by Anjani et al. (2021) [8].

It is worth noting that there was a significant difference in the diffusion efficiency of IBU sodium between the PVA-based H3 and H4 films after 24 h (*p* < 0.05). Specifically, the permeation of IBU sodium across H3 was greater than H4. Considering the swelling properties of H3 and H4, this result can be attributed to the high swelling degree of H3. Its lower crosslinked density and more porous hydrogel network allow more solutes to permeate across the swollen films into the receptor compartment [8, 39, 40]. Consequently, the hydrogel-forming MN arrays fabricated from H3 were combined with IBU sodium-loaded reservoirs for further ex vivo permeation studies.

### Fabrication and characterisation of hydrogel-forming MN arrays

Both digital and SEM images of the formed hydrogel-forming MN arrays are displayed in FigS1A-L. All MN arrays demonstrated a homogeneous physical appearance, with sharp needle tips, essential for skin insertion. The percentage height reduction for different MN array formulations was calculated and illustrated in FigS1M. Following a compression force of 32 N, H3 resulted in the greatest percentage height reduction (*p* < 0.05). This can be explained by the fact that the materials of H3 become softer and more elastic as a result of the shortened crosslinking time [8], resulting in greater bending after compression. However, statistical analysis indicated no significant difference in the percentage of height reduction for the other formulations (H1, H2 and H4) (*p* > 0.05). In general, all formulations showed no major loss in height, exhibiting appropriate mechanical strength for skin insertion, with a needle height reduction of less than 15% observed [8]. Moreover, all MN array formulations were able to successfully insert into the first three layers of Parafilm^®^ M, as presented in Fig S1N**.** Since each layer of Parafilm^®^ M has the mean thickness of approximately 125 μm, it can be inferred that all MN arrays are capable of penetrating to a depth of 378 to 504 μm, corresponding to 63–84% of the total MN height. The results confirm that all tested MN array formulations can penetrate the SC and reach the dermis, which offers a rich microvasculature system, ultimately permitting systemic drug delivery [5]. Additionally, Fig S1O-R displays the OCT images of MN arrays prepared from H1-H4. The MN arrays showed an insertion depth of approximately 447 μm, 456 μm, 426 μm and 413 μm into full-thickness neonatal porcine for H1, H2, H3 and H4 respectively. Statistically, no significant difference was found in the insertion depth of all formulations (*p* > 0.05). The results of this study align with those obtained previously [8].

### Preparation and characterisation of lyophilised reservoirs

**Fig. 5:**
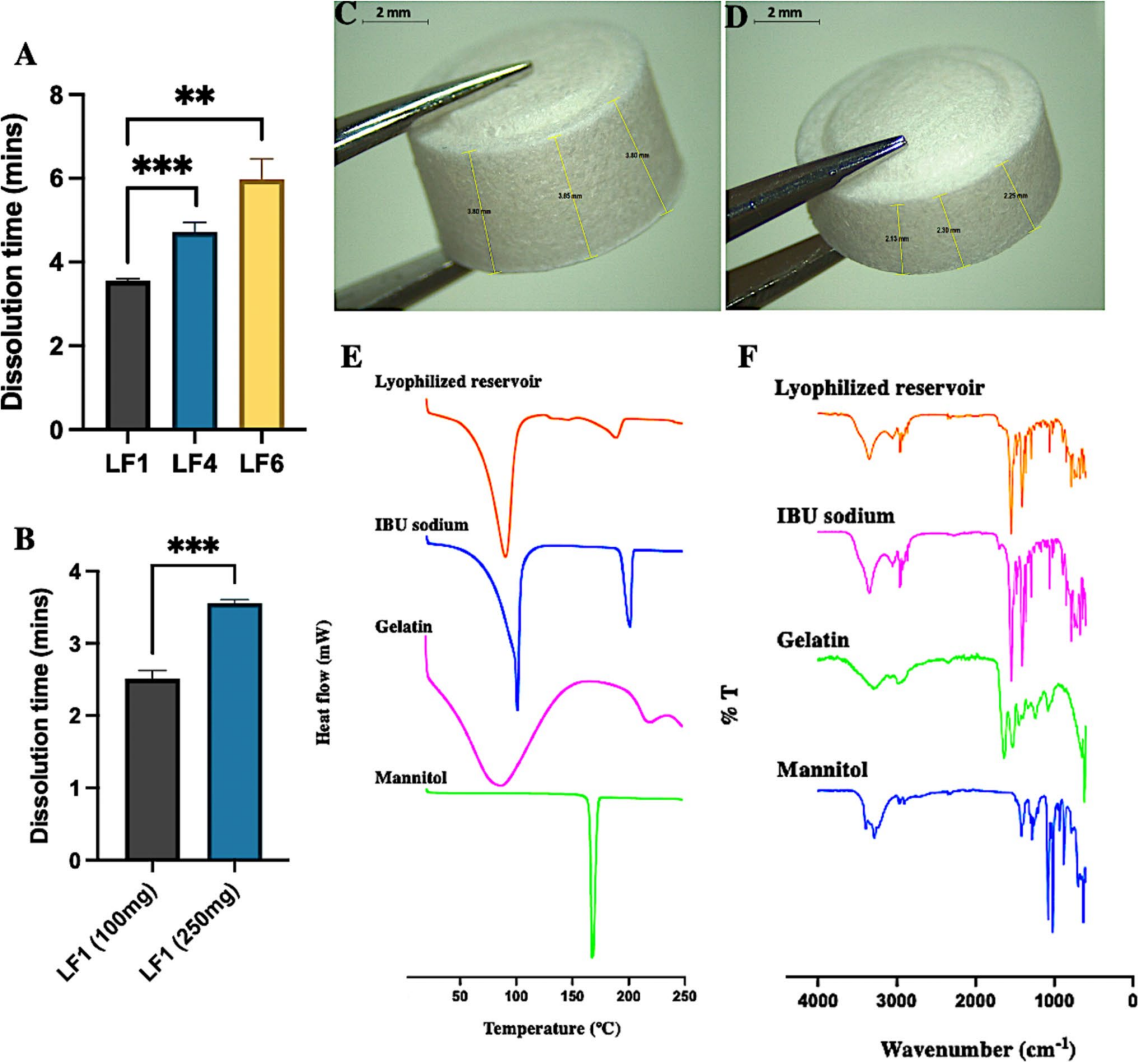
Dissolution time (in mins) of (**A**) LF1, LF4 and LF6 lyophilised reservoirs (**B**) LF1 (100 mg) and LF1 (250 mg) lyophilised reservoirs after being placed in a 25 mL PBS (pH 7.4), preheated to 37 °C, and stirred at 600 rpm using a stirring bar. (Means + SD, *n* = 3). (**p < 0.01, ***p < 0.001). Microscopic image of the variation in the thickness of the lyophilised reservoirs (**C**) LF1 (250 mg) (**D**) LF1 (100 mg). Representative (**E**) DSC thermograms (F) IR spectra of pure drug, LF1 lyophilised reservoirs and pure excipients, including gelatin and mannitol

The lyophilised reservoirs prepared from LF1, LF4, and LF6 were well-formed, robust, and homogeneous. However, LF2, LF3, and LF5 showed cracks, likely due to the lower %w/w of gelatin compared to LF1, LF4, and LF6. Reduced gelatin content results in fewer inter-chain hydrogen bonds, forming an unstable 3D network and lowering mechanical strength [46]. Thus, LF1, LF4, and LF6 were selected for further studies. The characterisation results are in Table S1. LF1 had the shortest dissolution time of 3.55 min compared to LF4 and LF6 (*p* < 0.05) (Fig. 5A), which may be attributed to the reduced water proportion in LF4 and LF6, which limits pore structure formation, resulting in slower dissolution. Higher crystalline bulking agent (mannitol) in LF4 also contributes to longer dissolution time [14, 47]. Moreover, to further improve dissolution, LF1 was made thinner (100 mg vs. 250 mg) as displayed in Fig. 5C-D, reducing dissolution time to 2.5 min (*p* < 0.05) (Fig. 5B). Recovery percentages of IBU sodium-containing reservoirs were 90–97%, showing stability of IBU sodium and no compatibility issues with excipients. All reservoirs met European Pharmacopoeia (2008) requirements in terms of the weight variation, hardness and friability [48]. Considering dissolution results, LF1 (100 mg) and LF1 (250 mg), consisting of 3% w/w mannitol, 10% w/w gelatin and 40% w/w IBU sodium, were selected for further studies due to their shorter dissolution times and higher drug loading.

The thermograms of pure IBU sodium, lyophilised reservoirs of LF1 and the pure excipients were depicted in Fig. 5E. A sharp endothermic peak was observed at 101℃ for pure IBU sodium at the temperature corresponding to its melting point, in which IBU sodium transferred from a solid powder state to a liquid state [49]. Meanwhile, there was another endothermic peak at 201℃, representing the thermal decomposition of IBU sodium. This was divided into three stages, namely dehydration, C-C bond breaking and intramolecular chemical bond breaking [50, 51]. It is noteworthy that although there were two endothermic peaks in the DSC thermogram of the IBU sodium-containing lyophilised reservoir, which were similar to those of pure IBU sodium, in all cases, the melting temperatures have been slightly shifted to lower temperatures and the peaks were slightly broader than those obtained from the pure drug. These results indicated that the crystallinity reduced during the lyophilization process [52, 53]. Moreover, for FITR analysis (Fig. 5F), the functional groups of pure IBU sodium were characterised by FTIR spectra peaks 853 cm^−1^ (C-H stretching), 1281 cm^−1^ (C-O stretching), 1543 cm^−1^ (C = O stretching) and 2949–3321 cm^−1^ (C-H stretching), which did not experience marked changes compared to LF1 lyophilised reservoirs, indicating that there are no compatibility issues between the excipient and the drug.

### Preparation and characterisation of effervescent reservoirs

**Fig. 6:**
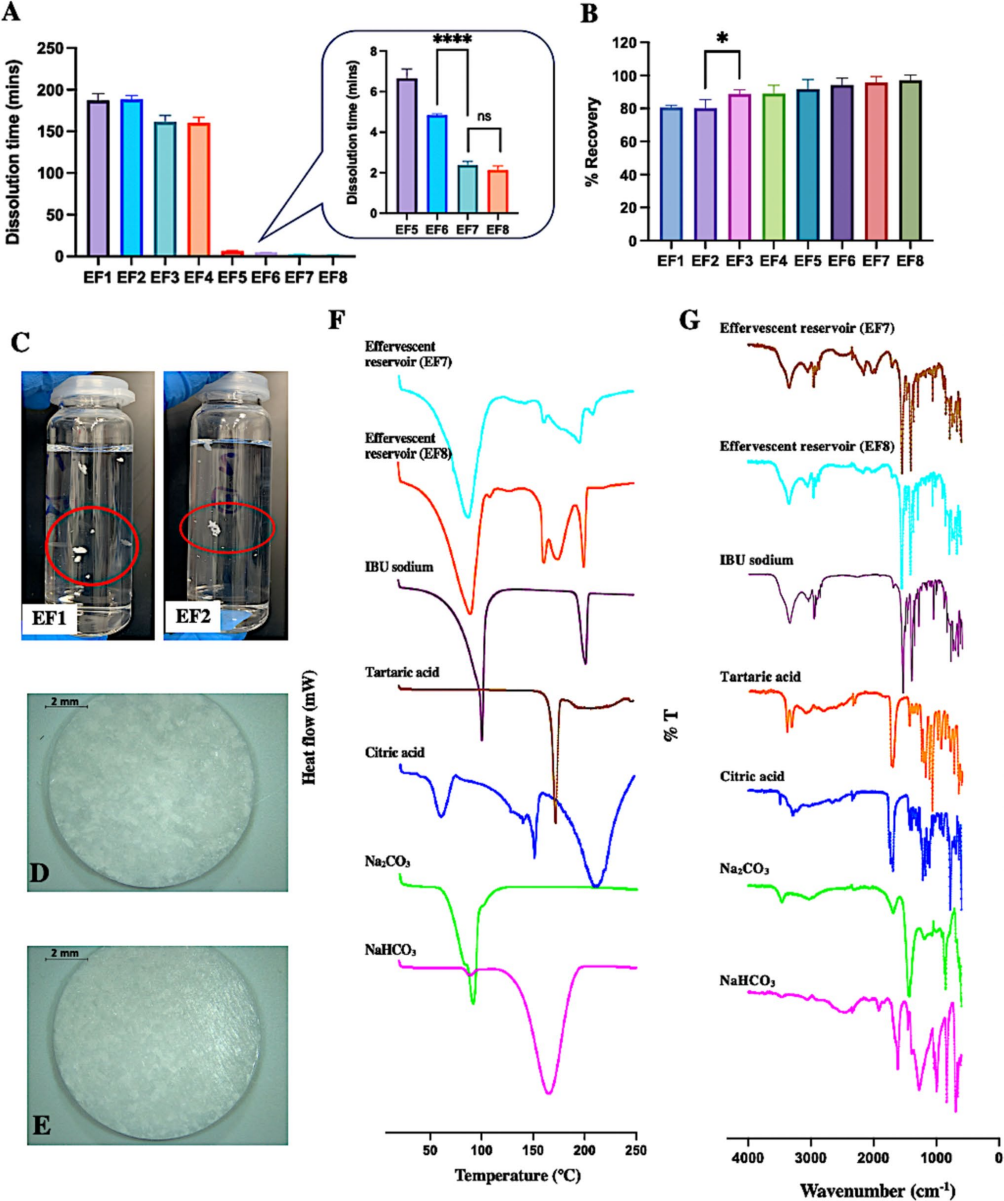
(**A**) Dissolution time (in mins) of different effervescent reservoirs after being placed in a 25 mL PBS (pH 7.4), preheated to 37 °C, and stirred at 600 rpm using a stirring bar. (Means + SD, n  = 3) (**** p  < 0.0001). (**B**) Percentage IBU sodium recovered from different effervescent reservoirs. (Means + S.D., n  = 3) (* p  < 0.1). (**C**) The dissolution of effervescent reservoirs from EF1 and EF2 after being placed in the incubator at 37℃ overnight. The microscopic image of effervescent reservoirs from (**D**) EF7 (**E**) EF8. Representative (**F**) DSC thermograms and (**G**) IR spectra of pure drug, representative of effervescent reservoir formulations (EF7 and EF8) and pure disintegrants, including citric acid, tartaric acid, Na _2_ CO _3_ and NaHCO _3._

In this study, the acid sources (citric acid and tartaric acid) and alkali metal carbonates (Na_2_CO_3_ and NaHCO_3_) act as disintegrants to cause rapid dissolution of effervescent tablets. The physical appearance of all formulations of effervescent tablets was well-formed and presented the characteristics of smooth surfaces, uniform colour, and no cracks. Therefore, dissolution and recovery studies were performed to further screen different formulations and the results were presented in Fig. 6A-B and Table S2. Given the rapid swelling of hydrogel-forming MN arrays upon insertion into the skin, a short dissolution time of less than 10 min is considered suitable for drug reservoirs [54]. The 1:1 acid-base neutralization ratio in EF1-EF4 resulted in an unsatisfactory dissolution time of over 1 h, significantly longer than other formulations (*p* < 0.05). This was due to the formation of water-insoluble IBU during the process. Kierys et al. (2017) found that acidic catalysts can lead to the conversion of IBU sodium into sodium chloride and a poorly soluble propionic acid derivative [45], IBU. Ha and Paek (2021) also reported that IBU sodium can be acidified to produce IBU [44]. Therefore, in EF1-EF4, IBU sodium reacted with citric or tartaric acid, producing poorly soluble IBU and slowing dissolution. Moreover, it was found that reducing the acid sources from 35 to 10% in EF5-EF8 could reduce the generation of IBU while retaining some effervescence, significantly shortening dissolution times (*p* < 0.05) to under 7 min. Among them, EF7 and EF8 dissolved in 2.37 and 2.12 min, respectively, much faster than EF5 and EF6 (*p* < 0.05). This was due to the increased proportion of Na_2_CO_3_ under the premise of maintaining the overall proportion of alkali carbonate at 50%. Na_2_CO_3_ has greater alkalinity than NaHCO_3_, resulting in enhanced alkalinity in EF7 and EF8, thus promoting the neutralization with acid and inhibiting the IBU formation. In recovery studies, EF1 and EF2 had recovery percentages of only 80%, significantly lower than other formulations (*p* < 0.05). Insoluble IBU was observed suspended in vials after overnight incubation at 37℃ (Fig. 6C), indicating precipitation exceeding saturation solubility. Increased Na_2_CO_3_ content in EF3 and EF4 improved recovery percentages to below 90%, better than EF1 and EF2 (*p* < 0.05). Besides, the recovery percentages from EF5-EF8 investigated were between 91 and 98% of the theoretical IBU sodium content, indicating relative drug stability in these formulations. Therefore, EF7 and EF8 (Fig. 6D-E), with the shortest dissolution times, acceptable recovery percentages, and compliance with pharmacopoeial requirements for hardness and weight variation (Table S3**)** [48], were selected for subsequent ex vivo permeation studies.

Figure 6 F shows the thermogram profiles of IBU sodium-containing effervescent reservoirs. The melting temperature of IBU sodium in effervescent reservoirs was slightly reduced, as well as its peak was slightly broader than pure IBU sodium. These results may be possibly due to the powder compaction during the compression process, which increased the density of IBU sodium, leading to intermolecular interactions and modification of crystal state [55]. The FTIR spectra is illustrated in Fig. 6G. Compared to both representative effervescent formulations, there were no marked changes observed in all characteristic functional groups of the pure drug. Hence, there were no compatibility issues with disintegrants.

### Preparation and characterisation of IBU sodium-loaded films

**Fig. 7:**
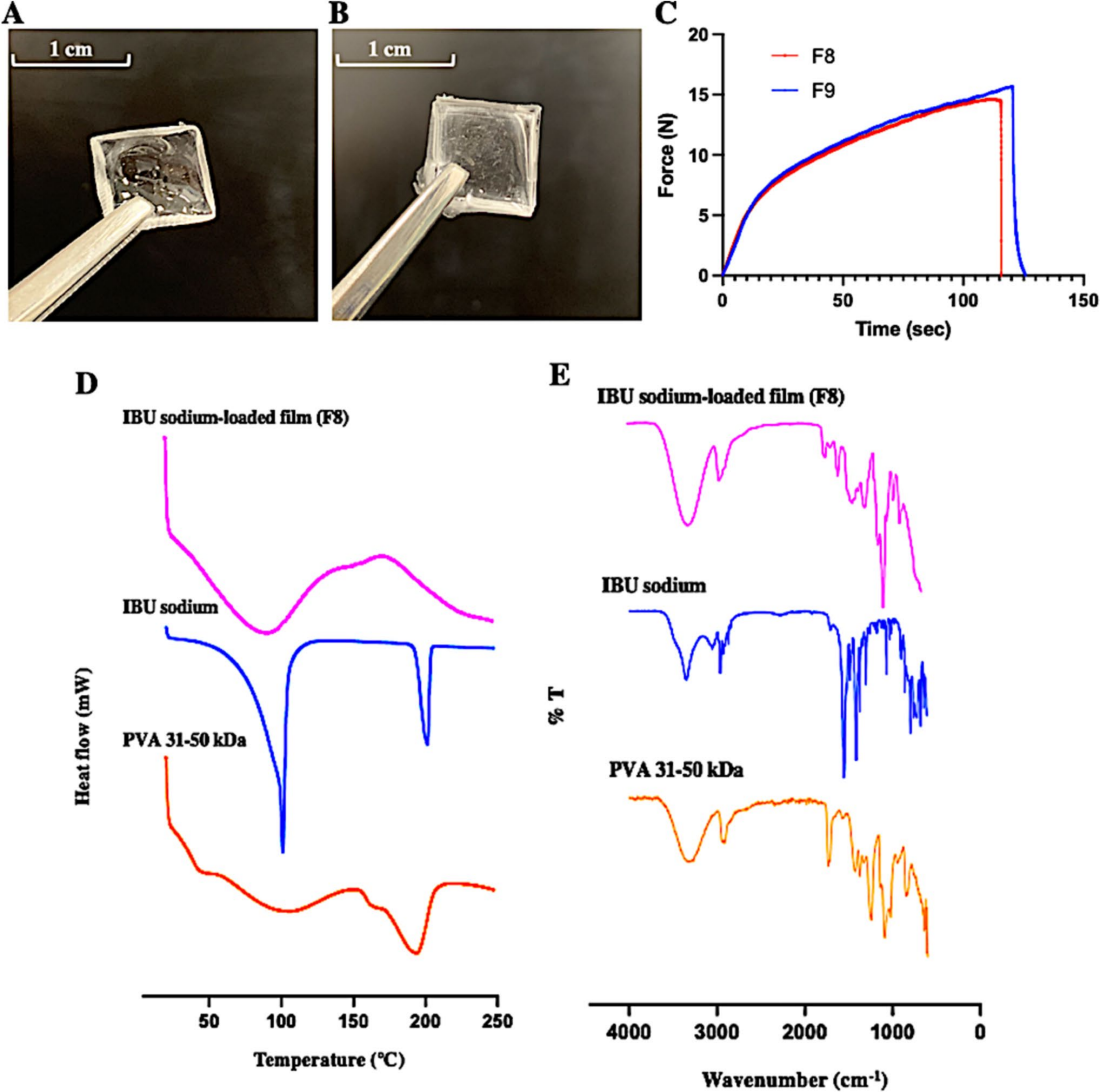
The digital images of IBU sodium-loaded films prepared from (**A**) F8 (**B**) F9 after casting from the 10 mm×10 mm square silicone moulds. (**C**) Typical tensile load/time profiles of IBU sodium-loaded films from F8 and F9. Representative (**D**) DSC thermograms (**E**) FTIR spectra of the pure drug, F8 IBU sodium-loaded film formulation and pure PVA 31–50 kDa

Based on the physical appearance, F8 and F9 were chosen for further screening in subsequent characterisation studies for their well-formed, elegant, flexible, uniform and clear physical appearance (Fig. 7A-B). Compared to F9 with a dissolution time of 9.48 min, F8 had a significantly shorter dissolution time of 7.81 min (*p* < 0.05). This may be explained by the increase in the content of IBU sodium in F9 compared to F8, which enhanced the hydrogen bonding interaction between the carbonyl groups in IBU sodium and the hydroxyl groups of PVA and glycerol, inhibited the interaction between PVA and glycerol with water molecules, and thereby prolonged the dissolution time of the IBU sodium-loaded films [56–58]. Moreover, the recovery percentages of F8 and F9 were 94.57% and 95.08%, respectively, which demonstrates the stability of IBU sodium in the films. Furthermore, mechanical strength studies showed that both F8 and F9 have certain flexibility and can withstand certain strain before fracture, with tensile strength of 4.37 and 4.68 N/mm^2^, and percent elongation at break of 250.99% and 262.67%, respectively. Meanwhile, Fig. 7C displays the typical tensile load/time profiles of F8 and F9, which shows that the two formulations had similar curves. Based on the previous results, F8, consisting of 15% w/w PVA 31–50 kDa, 15% w/w glycerol and 5% w/w IBU sodium, had the shortest dissolution time, as well as acceptable percentage recovery and mechanical strength, and was therefore chosen for the following ex vivo permeation studies combining with ITP and heat-assisted technology. Additionally, for DSC analysis (Fig. 7D), the melting peak of the IBU sodium-containing film was lower than the melting temperature of pure IBU sodium (101 °C), which is possibly due to the presence of the polymer in the film, leading to a decrease in the IBU sodium melting point [59]. Compared to the sharp peak of the pure drug, the endothermic peak of the film was broader, which can be explained by a certain degree of drug-polymer miscibility that resulted in reduced drug crystallinity [59]. Moreover, from the FTIR spectrum showed in Fig. 7E, the characteristic spectra of the IBU sodium-loaded films were different with the pure drug, with some peaks showing remarkable shifts. However, the main characteristic bands of PVA 31–50 kDa were similar to those observed in the film, which may be the result of the formation of intermolecular bonds between IBU sodium and PVA 31–50 kDa [60].

### Heating properties of the heat-generating powder mixture

The heat-assisted technology utilised in this study is based on the oxidative corrosion of iron powder in moist air, which generates heat. However, due to the slow oxidation process of iron powder under natural conditions, in addition to iron powder and water, activated carbon and sodium chloride (NaCl) are commonly added to many known thermal patches [61], which can form galvanic cell systems to accelerate the oxidation process. Iron powder and carbon serve as the cathode and anode, respectively, while NaCl forms an electrolyte solution with water, and then, with the participation of oxygen, chemical energy is converted into heat energy [62, 63]. The reactions that take place are as follows (Eqs. 5–8):

5 $$\mathrm{At}\;\mathrm{cathode}:\;\mathrm{Fe}\;-\;2\mathrm e^-\;=\;\mathrm{FE}^{2+}$$

6 $$\mathrm{Atanode}:\mathrm O^{2\;}+\;2{\mathrm H}_2\mathrm O\;+\;4\mathrm e^-\;=\;40\mathrm H^-$$

7 $$\mathrm{Total}\;\mathrm{reaction}:\;2\mathrm{Fe}\;+\;\mathrm O^2\;+\;2{\mathrm H}_2\mathrm O\;=2\mathrm{Fe}{(\mathrm{OH})}_2$$

8 $$4\mathrm{Fe}{\left(\mathrm{OH}\right)}_2\;+\;2{\mathrm H}_2\mathrm O\;+\;\mathrm O^2=4\mathrm{Fe}{(\mathrm{OH})}_3\rightarrow{\mathrm{Fe}}_2{\mathrm O}_3\cdot\mathrm{nH}{}_2\mathrm O$$

In addition, it has been reported that instead of using pure iron powder in most heating patches, it is most ideal to utilise iron powder of cast iron, pig iron, etc., containing carbon in the iron powder [64]. Where malleable iron with a carbon content of 3–4% is used, no carbon powder is required to be added. Moreover, pig iron powder is a solid solution with carbon, which prevents the generation of agglomerates during the mixing process of iron powder and carbon powder, thus avoiding partial limitation of the reactive surface of iron powder [65] Therefore, in this study, heat-generating powder mixtures were prepared from different formulations by altering the amount of pig iron powder (containing 3% carbon), deionised water and NaCl. The different formulations of the heat-generating powder mixtures are shown in Table 6. The heat properties of the heat-generating powder mixture were evaluated in terms of the maximum heating temperature and duration of temperature above 37℃, which are also recorded in Table 6.

**Table 6:** The heating temperature and duration of different heat-generated powder mixture formulations

Heating formulation	Formulation composition (g)			Heating properties	
	Pig iron powder (Contain 3% carbon)	NaCl	Water	Maximum heating temperature (℃)	Duration time (min)
HF1	1	0.05	0.05	33.1	−
HF2	1	0.1	0.05	33.1	−
HF3	1	0.15	0.05	33.2	−
HF4	1	0.2	0.05	33.9	−
HF5	1	0.25	0.05	34.3	−
HF6	2	0.1	0.1	38.1	7.74
HF7	2	0.2	0.1	38.17	8.15
HF8	2	0.3	0.1	38.73	10.16
HF9	2	0.4	0.1	38.9	10.75
HF10	2	0.5	0.1	39.47	12.35
HF11	2	0.1	0.2	35.7	−
HF12	2	0.2	0.2	35.5	−
HF13	2	0.3	0.2	32.2	−
HF14	3	0.2	0.1	41.17	19.83
HF15	3	0.3	0.1	42.07	22.98
HF16	3	0.4	0.1	42.53	24.4
HF17	3	0.5	0.1	43.2	27.09

A direct comparison between HF6-HF10 and HF11-HF13 revealed an increase in water content is accompanied by a significant reduction in heating temperature (*p* < 0.05). This can be explained by the fact that the increase in water molecules leads to less space between the particles of the substances. Consequently, there is a reduced surface area of iron powder exposed to oxygen, resulting in less heat generation [64]. Furthermore, while the content of iron powder and water in the heating formulations (HF6-HF10 and HF14-HF17) remained constant, the heating temperature significantly increased with increasing NaCl content (*p* < 0.05), as depicted in Fig S2A. This may be due to the higher concentration of electrolyte solution formed by NaCl and water, resulting in more ions in the solution that allow more redox reactions to occur [66]. Similarly, as the main component of heat, increasing the iron powder content also led to a significant rise in the heating temperature (*p* < 0.05). Besides, it is noteworthy that there was a significant prolongation of heating duration with increasing iron powder and/or NaCl content (*p* < 0.05) in HF6-HF10 and HF14-HF17, as displayed in Fig S2B. This can be attributed to the higher temperature, which took longer to cool to 37℃ than lower temperatures in the case of the same heat dissipation rate. During local skin heating, the skin temperature directly contributes to the control of local blood flow of the skin [67]. According to a study on the relationship between local skin temperature and forearm blood flow, skin blood flow increased slightly between 20℃ and 35℃, significantly at 37℃ and above, and maximally at about 42℃ [68]. Other studies have reported similar observations as well [69, 70]. A later study confirmed that vasodilation in the skin reached its maximum when the skin temperature was kept at 42℃ [71]. In the meantime, enhanced blood flow to the skin can facilitate drug absorption [20, 21], as a result, a skin temperature of 42℃ is considered optimal for transdermal drug absorption. Therefore, although both HF15 and HF16 exhibited an optimal heating temperature of approximately 42℃, the longer duration of HF16 makes it a more suitable candidate for further ex vivo permeation studies combining the heat-generating technology with integrated hydrogel-forming MNs.

### Ex vivo permeation studies of lyophilised and effervescent reservoirs

**Fig. 8:**
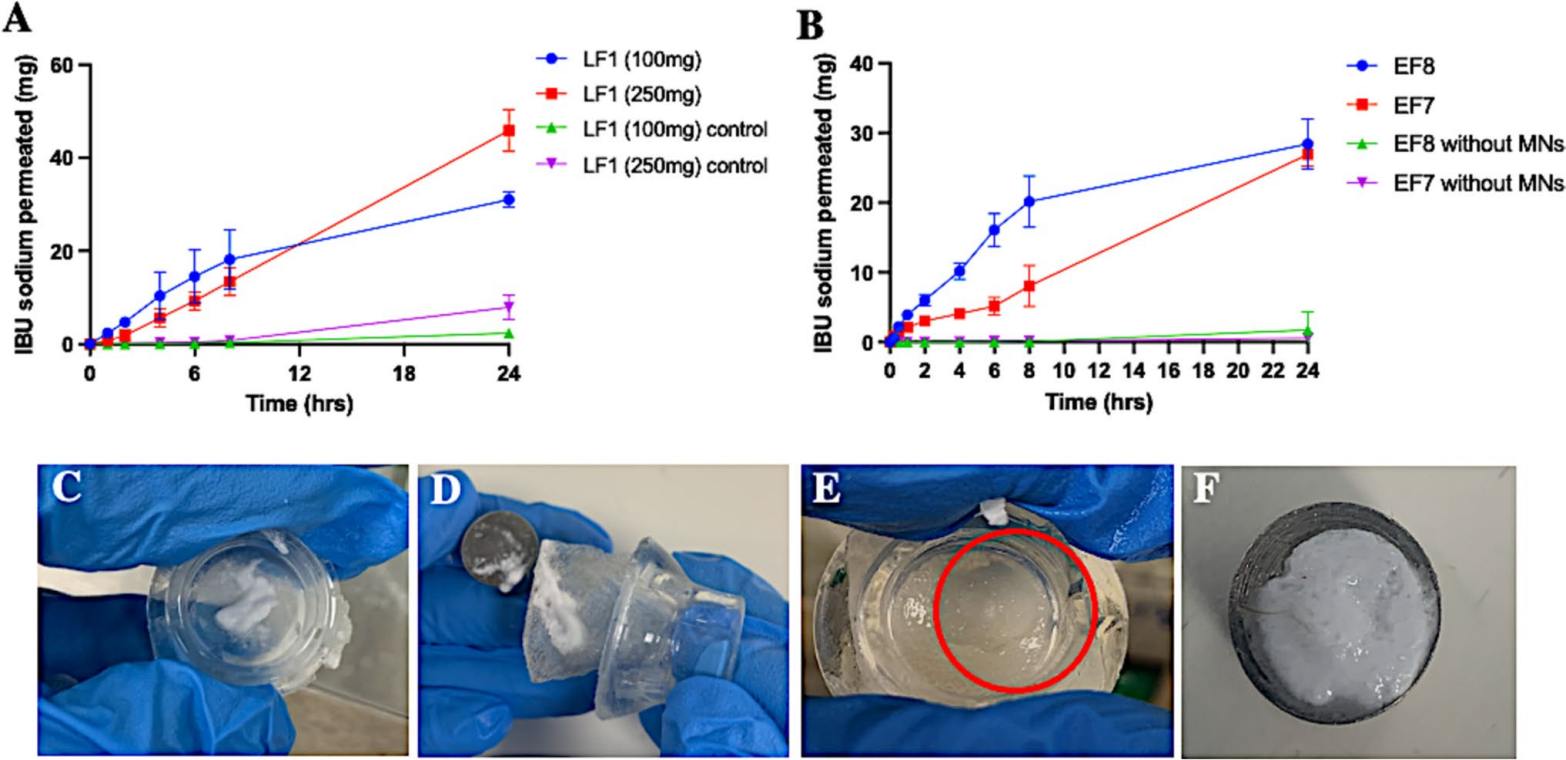
Ex vivo permeation profiles of IBU sodium from (**A**) lyophilised reservoirs, (**B**) effervescent reservoirs when combined with hydrogel-forming MN arrays. (Means + SDs, *n* = 3). The residuals of the lyophilised reservoir of (**C**) LF1 (100 mg), (**D**) LF1(250 mg) and effervescent reservoirs of (**E**) EF7, (**F**) EF8 after 24 h without hydrogel-forming MN-mediated drug delivery

The drug permeation profiles of LF1 (100 mg) and LF1 (250 mg) lyophilised reservoirs across dermatomed neonatal porcine skin over 24 h are presented in Fig. 8A. The results showed that IBU sodium exhibited a high permeation efficiency from LF1 (100 mg) lyophilised reservoirs, with approximately 78% of the theoretical loading of IBU sodium, equivalent to 30.66 mg, delivered after 24 h, of which 45% (equal to 16.65 mg) of IBU sodium had already permeated into the receptor compartment before the 8 h timepoint. This rapid and highly efficient permeation can be explained by the porous structure of lyophilised reservoirs and its mostly hydrophilic components, leading to fast disintegration. In addition, only 46% (equal to 45.88 mg) of IBU sodium was delivered from the lyophilised reservoirs of LF1 (250 mg) after 24 h, which was significantly lower than LF1 (100 mg) (*p* < 0.05). Also, the release profile of LF1 (250 mg) was relatively constant compared to LF1 (100 mg). This can be attributed to the fact that, as mentioned above, the dissolution time of LF1 (250 mg) was much longer than that of LF1 (100 mg) (*p* < 0.05), so the lyophilised reservoirs of LF1 (250 mg) may not be completely dissolved within 24 h, thereby resulting in lower permeation. Furthermore, it is noticeable that the permeation profiles of both LF1 (250 mg) and LF1 (100 mg) did not achieve a plateau state after 24 h and the release curve still has an upward trend. Hence, it is feasible that the permeation of IBU sodium would continue to increase if the sampling time was extended.

In the case of effervescent reservoirs, as shown in Fig. 8B, IBU sodium from both effervescent reservoirs of EF7 and EF8 exhibited a high permeation efficiency after 24 h, with approximately 67% (26.93 mg) and 71% (28.43 mg) of the theoretical loading of IBU sodium being delivered to the receiver compartment, respectively. Interestingly, up to the 8 h time point, the effervescent reservoirs of EF7 and EF8 showed significantly different permeation efficiencies. By this time, approximately 50%, equivalent to 20.14 mg of IBU sodium in EF8, had already permeated across dermatomed neonatal porcine skin and into the receiver compartment. However, only 20% (8.06 mg) of IBU sodium was delivered from EF7 reservoirs within 8 h, which was significantly lower than EF8 (*P* < 0.05). Considering this further, in EF8, when the total proportion of acid sources was kept at a sufficiently low level of 10% to effectively inhibit the generation of IBU, the proportion of tartaric acid was increased. The acidity of tartaric acid is stronger than citric acid, so the use of tartaric acid in effervescent reservoirs had a greater effervescent effect and generated more CO_2_ bubbles, which can further promote the disintegration of effervescent tablets [72]. Moreover, the greater amount of CO_2_ bubbles generated can increase the surface pressure of hydrogel-forming MNs and accelerate the deep delivery of IBU sodium through swollen hydrogel MNs into the receiver compartment [16]. Therefore, the disintegration of EF8 was faster than EF7 in the early period, allowing drug molecules to be released from the reservoirs more quickly, resulting in early rapid delivery of EF8.

To investigate the effect of the presence of hydrogel-forming MNs on TDD, the control cells were also set up. The delivery efficiency of IBU sodium was significantly reduced in the absence of hydrogel-forming MNs for both lyophilised and effervescent reservoirs (*p* < 0.05). On the one hand, compared with the complete dissolution of the reservoirs cooperating with hydrogel-forming MNs, a large amount of reservoir residue was found in the control groups due to the lack of sufficient release medium in the donor provided by swollen hydrogel-forming MN arrays, as displayed in Fig. 8C-F. In addition, due to the absence of hydrogel-forming MNs, the *SC* barrier cannot be bypassed, making it increasingly difficult for IBU sodium to permeate [73]. In summary, both lyophilised and effervescent reservoirs were successfully developed as a rapidly dissolving reservoir. Among them, the higher permeation efficiency of LF1 (100 mg) lyophilised reservoirs and EF8 effervescent reservoirs were closer to the aim of rapid and efficient TDD. As a result, the two formulations were decided to be evaluated in vivo using a rat model.

### Ex vivo permeation studies of MN + ITP

**Fig. 9:**
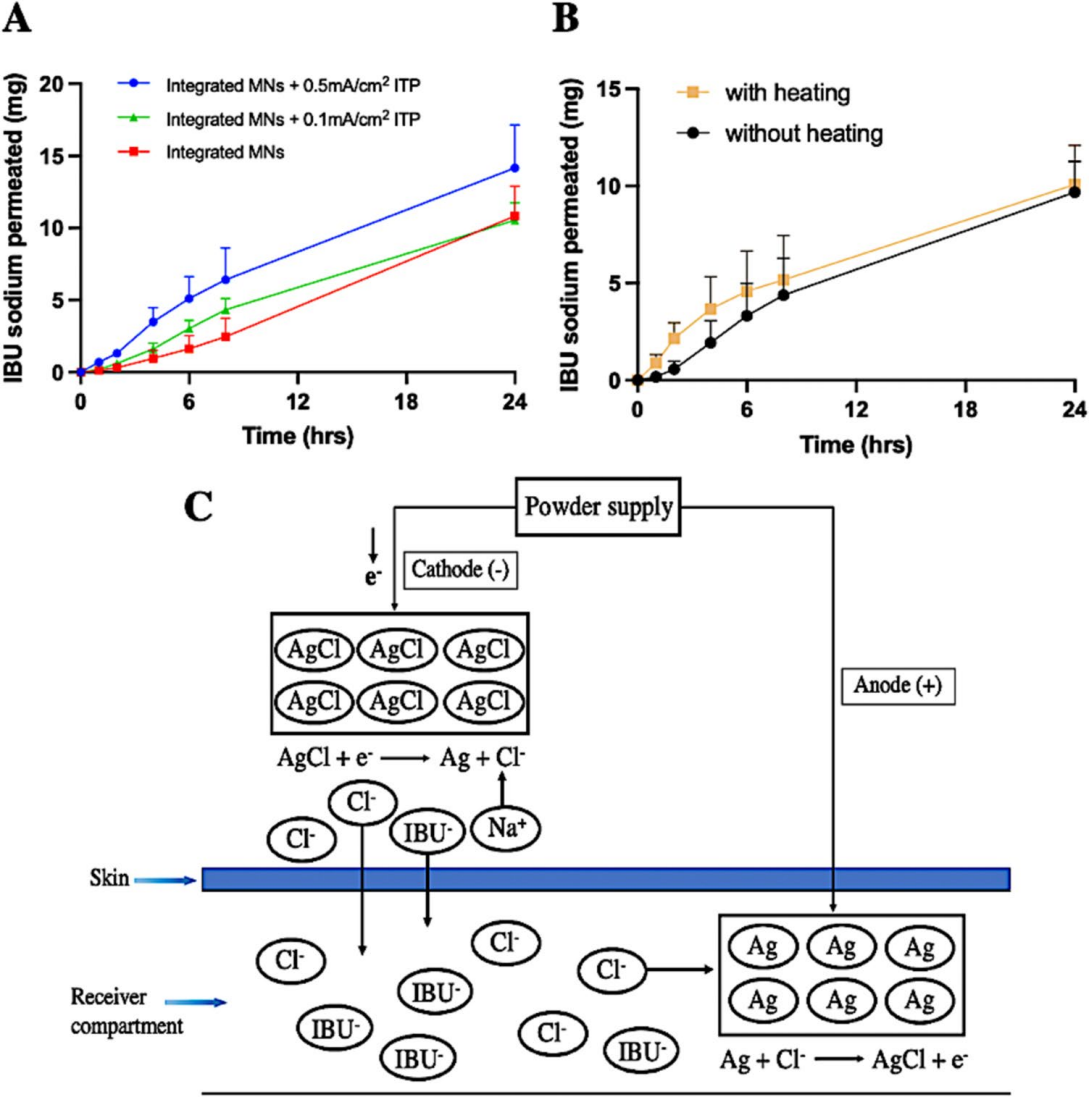
Ex vivo permeation profile of IBU sodium delivered by (**A**) MN + ITP strategy, (**B**) MN + heating strategy. (Means + SDs, *n* = 3). (**C**) Schematic illustration of the process of ITP in an Ag/AgCl electrode system driving transdermal absorption of IBU sodium

Figure 9A illustrated the ex vivo permeation profiles over a 24 h study period, with IBU sodium delivered following ITP at a current density of 0.1 mA/cm^2^ or 0.5 mA/cm^2^ for 6 h. In general, the combination of ITP with a current density of 0.5 mA/cm^2^ and integrated hydrogel-forming MNs achieved a greater rate and overall extent of TDD compared with the integrated MNs alone. After 24 h, in comparison to approximately 43% (10.82 mg) of the theoretical loading of IBU sodium delivered by integrated MNs alone, approximately 57% (14.15 mg) of IBU sodium permeated into the receiver compartment when combined with ITP, however, the difference was not significant (*p* > 0.05). This may be related to the low MW of IBU sodium. Wu et al.. (2007) reported a related finding that although the transdermal penetration of high-MW compounds through MN-induced channels could be increased by combining with ITP [19], the penetration of low-MW compounds did not increase significantly, which were otherwise passively permeating to a high degree through the MN-induced channels. Meanwhile, the authors also evaluated the relationship between the MW and the permeability coefficient of the penetrant, pointing out that the contribution of ITP to enhancing drug permeation through MN-pretreated skin increased with increasing MW. Another study reported similar results [5].

It is important to note that in the early time points (0–6 h), the combination of ITP with 0.5 mA/cm^2^ current density and integrated MNs demonstrated a significantly higher permeation efficiency than integrated MNs alone (*p* < 0.05). After 6 h, approximately 20%, equivalent to 5.11 mg of IBU sodium, was delivered from the IBU sodium-loaded film when combined with ITP, whereas for the integrated MNs alone, only 6% (1.63 mg) of IBU sodium was detected in the receiver compartment, which was significantly lower than the MN + ITP delivery strategy (*p* < 0.05). The early period of 0–6 h coincides with the application time of ITP. According to the principle of ITP, in which like charges repel each other and unlike charges attract each other [74], when IBU sodium (a negatively charged drug) was to be delivered across the skin, it was placed under the cathode, which was repelled and attracted to the anode placed in the receiver compartment, as presented in Fig. 9C. Therefore, more IBU sodium was attracted to the receiver compartment in the early stages. In addition, in an in vitro experiment, OCT analysis by Donnelly et al.. (2012) revealed that the application of an electrical current causes a sharp increase in the swelling of hydrogel-forming MNs [5]. The author explains this result as an enhancement of water uptake by electroosmosis, which may also partially explain the significant improvement in drug permeation efficiency observed in the early period by MN + ITP delivery strategy in this study. Furthermore, the ability of ITP with a lower 0.1 mA/cm^2^ current density in combination with integrated MNs to accelerate TDD was also evaluated, but the results showed that there was no significant increase in IBU sodium permeation either in the early stage or 24 h later, which indicated that the magnitude of the applied current largely determined the drug delivery rate from an ITP system [75]. Similarly, Vemulapalli et al. (2008) reported that under prolonged application of ITP, an increase in current density increased the cumulative amount of methotrexate delivered [76]. Moreover, the clinical value of the normal maximum current density reported by Riviere et al.. (1995) is 0.5 mA/cm^2^, exceeding which local tissue damage and reduced blood flow may occur [77]. In summary, the current density had a certain effect on the skin permeation of IBU sodium. The MN + 0.1 mA/cm^2^ ITP delivery strategy did not exhibit any significant enhancement in TDD. However, although there was no significant improvement in permeation efficiency after 24 h with 0.5 mA/cm² ITP combination, the significantly increased drug delivery within the first 6 h still demonstrated potential for rapid drug delivery.

### Ex vivo permeation studies of MN + heat-assisted technology

In terms of heating, the results (Fig. 9B) showed that there was no significant difference between MN alone and MN + heating delivery strategy on the permeation efficiency of IBU sodium across dermatomed neonatal porcine skin (*p* < 0.05), with approximately 39% (9.68 mg) and 40% (10.08 mg) of the theoretical loading of IBU sodium delivered after 24 h, respectively. Contrastingly, by investigating the ex vivo permeation profiles in the early period, it was found that the amount of IBU sodium permeating from the MN + heating delivery strategy was significantly higher than MN alone at 2 h (*p* < 0.05). However, after that, there was no significant difference between the two strategies (*p* > 0.05), which may be related to several factors. Skin hydration is known to increase penetration [78, 79], while for ex vivo permeability studies, the hydration of the skin by a large amount of release medium in the receiver compartment led to higher permeation of IBU sodium. Therefore, in this instance combining heating technology with hydrogel-forming MN arrays does not further improve delivery. In addition, although the IBU sodium-containing films and hydrogel-forming MNs placed under the heat-generating device are thermally conductive, the thickness of the materials may result in heat loss during heat transmission from the heat-generating device to the skin below. In the meantime, during the experiment, the quantity of moisture from the swollen hydrogel-forming MNs may also lead to heat loss, resulting in a reduction of the permeation-promoting effect on IBU sodium when combined with heating technology. Moreover, the high hydrophilicity and low MW of IBU sodium are the attributes applicable to TDD, which induces high permeability and therefore leads to less temperature dependence [80]. Overall, there was no significant difference in the amount of IBU sodium delivered using the two different delivery strategies. However, given that heating does have an effect on drug penetration at the 2-h time point, and that its short duration (approximately 30 min) minimizes any effects over 24 h, the heating combination strategy still has research value. Meanwhile, extending the heating duration may be worth exploring in future studies.

It is crucial to consider the practicality of using complex multi-component drug delivery devices (MN + ITP/heat-assisted technology) for human applications. In terms of safety and patient compliance, studies have shown that skin irritation caused by ITP resolves spontaneously and rapidly, without permanent damage or disruption to the skin barrier [81, 82], confirming the safety of drug delivery *via* ITP. Regarding the heat-assisted strategy, research indicates that human skin can tolerate temperatures up to 42 °C (the maximum heating temperature in this study) for short periods without significant adverse effects, as this is below the threshold for protein denaturation or burns, which typically occurs around 44–45 °C [83]. Additionally, the minimal invasiveness of hydrogel MNs and the absence of polymer residue after application greatly enhance patient compliance. Furthermore, unlike previous bulky devices, wearable ITP therapy devices have become increasingly common over the past two decades [84, 85], allowing current values to be controlled via smartphones using Bluetooth, significantly improving convenience and quality of life. While further advancements are needed for wearable heat-assisted devices combined with MNs—specifically temperature monitoring and regulation to ensure patient safety and comfort during clinical use—these multi-component devices demonstrate promising potential for human applications.

### In vivo studies of lyophilised and effervescent reservoirs

**Fig. 10:**
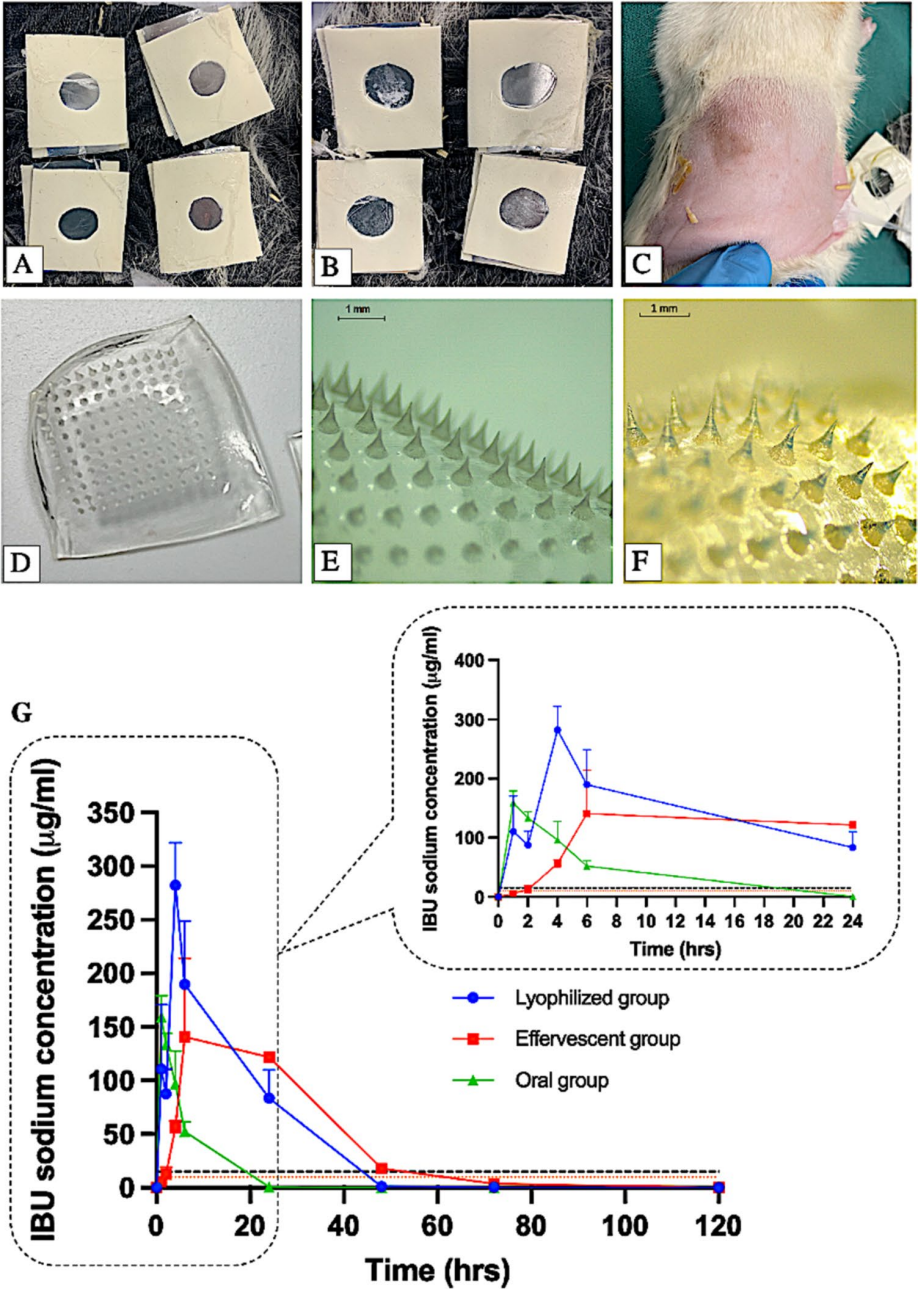
Digital and microscopic images taken 5 days following the application of drug reservoirs and hydrogel MNs to the rats and immediately upon removing them. (**A**-**B**) The site of application of lyophilised and effervescent reservoirs, respectively. (**C**) The removal of the swollen hydrogel-forming MN arrays from the rats’ backs completely intact without leaving any polymer behind or causing damage. (**D**) Swollen hydrogel-forming MN array upon its removal from the skin and backing layer. (**E**-**F**) Digital microscopic images of a swollen hydrogel-forming MN array following its removal from rat skin during the in vivo study in lyophilised and effervescent group, respectively. (**G**) IBU sodium plasma profiles of the rats in the oral, lyophilised, and effervescent groups following the in vivo study for 5 days. (Means + SDs, *n* = 3 at 1, 2, 4 and 6 h, *n* = 6 at the remaining time points). The black and orange dashed lines represent the human therapeutic plasma range of IBU sodium (10–15 µg/ml)

In the in vivo study, after the completion of sampling on Day 5, upon removal of the drug reservoirs and hydrogel MNs from Group 2 and 3, it was observed that all the drug reservoirs were completely dissolved and disappeared, with no observable residues remaining within the system, as shown in Fig. 10A-B. Furthermore, Fig. 10D-F shows the typical morphology of the hydrogel-forming MNs when removed from the rat skin after 5 days. This indicates that the MNs maintained their structural integrity throughout the application and extensively absorbed skin interstitial fluid (ISF) to form a swollen hydrogel matrix, thus facilitating the transdermal delivery of IBU sodium into the systemic circulation. The hydrogel-forming MNs exhibited sufficient robustness when swollen to ensure their intact removal, without breaking any tips or leaving any polymer residues in the skin. This is consistent with the findings reported previously [12, 86, 87], and confirms the versatility of the developed hydrogel-forming MNs. In addition, the application of MNs did not result in any significant damage to the rats’ skin, as illustrated in Fig. 10C.

The in vivo plasma profiles of IBU sodium from the rats in the three groups, namely, oral, lyophilised, and effervescent groups, are collated in Fig. 10G. It can be found that the plasma concentration of IBU sodium rapidly increased after oral administration. After 1 h, the plasma concentration reached its maximum of 159.17 µg/mL. Sequentially, it began to decline sharply, reaching a concentration of 52.3 µg/mL at 6 h before it decreased below the limit of quantification of the in vivo HPLC method (0.25 µg/mL) after 24 h. Compared to the oral group, the integrated hydrogel-forming MN systems applied in the Group 2 and 3 both exhibited more sustained plasma levels. This can be attributed to the 5-day wear time, during which IBU sodium is continually delivered by the MN system. During the swelling process upon application to the skin, the drug reservoir can dissolve by contacting the moisture in the hydrogel-forming MNs. Afterwards, the components of the drug reservoirs diffuse through the swollen hydrogel-forming MNs into the dermal microcirculation. Therefore, the time required for MNs to swell results in a longer time to complete dissolution of the drug reservoir. This attributes to the sustained IBU sodium release observed in this study. Moreover, unlike ex vivo permeation studies where a large amount of release media was contained in the receiver compartment of the Franz cell, there was limited ISF present in rat skin for in vivo studies, which also prolongs the dissolution time of drug reservoirs.

An extensive increase in the plasma concentration of IBU sodium over 6 h was observed in the effervescent group, with a maximum concentration of 140.81 µg/mL observed in this time. This value was not significantly different from the maximum plasma concentration of 282.15 µg/ml achieved at 4 h in rats receiving lyophilised reservoirs (*p* > 0.05). After 24 h, the IBU sodium plasma concentration of the rats from the lyophilised group significantly fell to 83.55 µg/mL (*p* < 0.05), while in case of the effervescent group, a relatively constant plasma concentration was maintained, being 140.81 µg/mL at 6 h and 121.64 µg/mL at 24 h. However, there was no significant difference in the value of plasma concentration between the two groups at this time point (*p* > 0.05). The plasma levels of both groups were then followed up to the 48-h time point. However, it is worth noting that at 48 h, the plasma concentration of the effervescent group was 17.89 µg/mL, which was significantly higher than the lyophilised group (*p* < 0.05), where the plasma concentration had already fallen below the quantification limit of the method (0.25 µg/mL). This may be explained by the generation of a small quantity of water-insoluble IBU due to the reaction between IBU sodium and acidic disintegrants in effervescent reservoirs. This may result in the accumulation of IBU in rat skin, thereby prolonging the period of drug release [45].

The therapeutic plasma level of IBU in the human body has been reported to range between 10 and 15 µg/mL [88]. These levels were achieved within the first hour and the second hour of MN application in the lyophilised and effervescent groups, respectively. Furthermore, while the therapeutic concentration can be reached within the first hours, at least 48 h of sustained transdermal delivery of IBU sodium can be achieved with the two systems. Therefore, the combination of lyophilised or effervescent reservoirs with hydrogel-forming MNs may effectively reduce administration frequency, enhance patient compliance and show fewer side effects [63]. Additionally, for patients with a phobia of swallowing tablets, integrated hydrogel-forming MNs are considered an alternative route of administration, ensuring painless and irritation-free drug delivery.

Moreover, in vivo plasma pharmacokinetic parameters (C_max_, T_max_ and AUC _0−t_) of IBU sodium from all three groups are presented in Table S4. The AUC _0−t_ values obtained from both the lyophilised and effervescent groups were similar, with values of 4493.5 µg/ml*h and 4611.72 µg/ml*h, respectively (*p* > 0.05). Additionally, compared to the oral group, a delay in T_max_ was observed in both lyophilised and effervescent groups, indicating sustained release. However, this delayed T_max_ is still shorter than the previously reported T_max_ of approximately 24 h when using hydrogel-forming MN arrays combined with other types of drug reservoirs to deliver small molecule, water-soluble drugs like esketamine and donepezil hydrochloride in rats [54, 89]. This suggests that both lyophilised and effervescent reservoirs showed reduced onset of action compared to earlier drug reservoir designs, enabling rapid TDD when combined with hydrogel-forming MNs.

Based on these results, the required patch size for human volunteer studies can be estimated using the body surface area (BSA) normalization method, as shown in Eq. 9 [90–93]. This allows for the extrapolation of data from pharmacokinetic studies in rats to estimate the appropriate patch size for human application:

9 $$\:HED\left(\frac{mg}{kg}\right)=AD\left(\frac{mg}{kg}\right)\times\:\left(\frac{rat\:\:{K}_{m}}{human\:{K}_{m}}r\right)$$

where HED is the human equivalent dose, AD is the animal dose, the rat K_m_ factor is 6, and the human K_m_ factor is 37 [94].

Considering that the therapeutic plasma level of IBU sodium *via* oral administration begins to provide pain relief at 5–10 µg/mL [95, 96], with a therapeutic range of 10–50 µg/mL [97, 98], this study demonstrated that an adult patient weighing 60 kg with a BSA of 1.6 m² would require approximately 400 mg of IBU sodium transdermally over a 5-day period using lyophilised or effervescent reservoirs. In the rat study, the C_max_ values were 282.15 µg/mL and 140.81 µg/mL for lyophilised and effervescent reservoirs, respectively. Extrapolating these amounts to human patch size, a 1 cm² patch would be sufficient to achieve therapeutic levels. This is relatively small compared to typical commercial transdermal patches, which can be as large as 30–40 cm² [12], such as the Duragesic CII (fentanyl) patches designed by Janssen, with areas of 32 and 42 cm² [99]. Thus, it is reasonable to propose that products combining both lyophilised and effervescent reservoirs with hydrogel-forming MNs could be effectively developed for pain management.

## Conclusion

The work presented in this article described the feasibility of different physical and chemical modification methods of drug reservoirs to accelerate the transdermal delivery of IBU sodium when combined with hydrogel-forming MN arrays. The ITP-mediated combination strategy demonstrated a significant enhancement of IBU sodium transdermal permeation over the first 6 h. Moreover, in terms of heat-assisted drug delivery technology, almost no effective promoting effect on transdermal delivery was observed. In the case of the two chemical modification methods, lyophilised and effervescent reservoirs, in vivo studies indicate that both strategies not only achieve rapid attainment of therapeutic levels suitable for acute pain, but also enable the sustained release of IBU sodium, which is beneficial for chronic pain management. Future work may involve exploring the universality of these two strategies for different drugs by selecting different model drugs, such as drugs with varying MWs.

## Supplementary Information

Below is the link to the electronic supplementary material.

ESM 1 (DOCX 5.79 MB)

## Data Availability

Data will be made available on request. All data are provided in the manuscript and supplementary file.
