# Supplementary material for: Evaluation of physical and chemical modifications to drug reservoirs for stimuli-responsive microneedles
Source: Drug Deliv Transl Res. 2024 Nov 20;15(7):2390–414. doi: 10.1007/s13346-024-01737-0 (PMC12137397; doi:10.1007/s13346-024-01737-0)
Supplement: Supplementary file 1 — (DOCX 5.79 MB) [file 13346_2024_1737_MOESM1_ESM.docx]

Supplementary Data

Evaluation of physical and chemical modifications to drug reservoirs for stimuli-responsive microneedles

Luchi Li^a^, Qonita Kurnia Anjani^a^, Aaron R. J. Hutton^a,b^, Mingshan Li^a^, Akmal Hidayat Bin Sabri^a^, Lalitkumar Vora^a^, Yara A. Naser^a^, Yushi Tao^a^, Helen O. McCarthy^a^, Ryan F. Donnelly^a,*^.

*^a^ School of Pharmacy, Queen's University Belfast, Medical Biology Centre, 97 Lisburn Road, Belfast BT9 7BL, UK*

*^b^ School of Pharmacy and Pharmaceutical Sciences, Ulster University, Pharmacy Building, Block Y, 1SA, Cromore Rd, Coleraine BT52 1SA, UK*

*Corresponding author at: Chair in Pharmaceutical Technology, School of Pharmacy, Queen’s University Belfast, Medical Biology Centre, 97 Lisburn Road, Belfast BT9 7BL, Northern Ireland, UK

E-mail: [r.donnelly@qub.ac.uk](mailto:r.donnelly@qub.ac.uk) (R.F. Donnelly)

**Table S1.** Characterisation results of IBU sodium lyophilised reservoirs (means ± SD, n = 3).

| **Formulation** | **Dissolution time (mins)** | **Recovery (%)** | **Hardness**  **(N)** | **Friability (%)** | **Weight**  **(mg)** |
| --- | --- | --- | --- | --- | --- |
| LF1 (100 mg) | 2.51 | 97.38 ± 2.01 | 45.25 ± 2.5 | 0.6% | 55.3 ± 2.35 |
| LF1 (250 mg) | 3.55 | 92.68 ± 0.94 | 83.25 ± 1.7 | 0.3% | 133.63 ± 2.33 |
| LF4 | 4.72 | 90.12 ± 0.84 | 112 ± 1.41 | 0.05% | 142.88 ± 2.13 |
| LF6 | 5.97 | 91.04 ± 0.94 | 78.75 ± 3.77 | 0.1% | 145.9 ± 1.41 |

**Table S2.** % recovery and dissolution time for different effervescent reservoir formulations. (means ± SD, n = 3).

| **Formulation** | **Theoretical drug content (mg)** | **% Recovery** | **Dissolution time (mins)** |
| --- | --- | --- | --- |
| EF1 | 30 | 80.60 ± 1.38 | Over 1 hour |
| EF2 | 30 | 80.17 ± 5.13 | Over 1 hour |
| EF3 | 30 | 88.81 ± 2.56 | Over 1 hour |
| EF4 | 30 | 89.03 ± 5.06 | Over 1 hour |
| EF5 | 40 | 91.79 ± 5.78 | 6.65 |
| EF6 | 40 | 94.25 ± 4.12 | 4.84 |
| EF7 | 40 | 95.75 ± 3.56 | 2.37 |
| EF8 | 40 | 97.18 ± 3.65 | 2.12 |

**Table S3.** Physical evaluation parameters of IBU sodium effervescent reservoirs. (means ± SD, n = 3).

| **Formulation** | **Parameter** | | |
| --- | --- | --- | --- |
|  | **Thickness (mm)** | **Hardness (N)** | **Weight (mg)** |
| EF7 | 0.81 ± 0.017 | 30.25 ± 1.893 | 99.36 ± 0.488 |
| EF8 | 0.80 ± 0.018 | 32 ± 1.826 | 99.57 ± 0.463 |

**Table S4.** *In vivo* plasma pharmacokinetic parameters of IBU sodium for oral, lyophilised, and effervescent groups. (Means ± SDs, n = 6 for each group).

| **Parameter** | **Unit** | **Control (oral)** | **Lyophilised group** | **Effervescent group** |
| --- | --- | --- | --- | --- |
| **T _max_** | Hour | 1 | 4 | 6 |
| **C _max_** | µg/ml | 159.17 ± 34.79 | 282.15 ± 68.68 | 140.81 ± 127.28 |
| **AUC _0-t_** | µg/ml*h | 1089.43 ± 193.66 | 4493.5 ± 44.58 | 4611.72 ± 1241.15 |

**Table S5.** Light microscope and digital images of LF1-LF6 lyophilised reservoir formulations.

| **Formulation** | **Microscopic image, 8x magnification** | **Comments** |
| --- | --- | --- |
| LF1 | 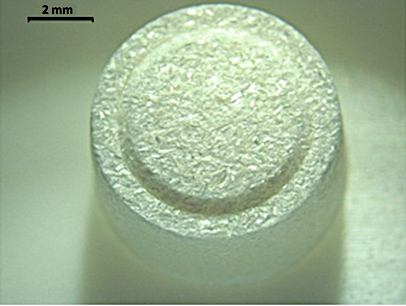 | Well-formed, robust, elegant and homogeneous reservoir |
| LF2 | 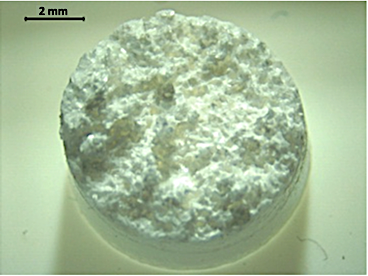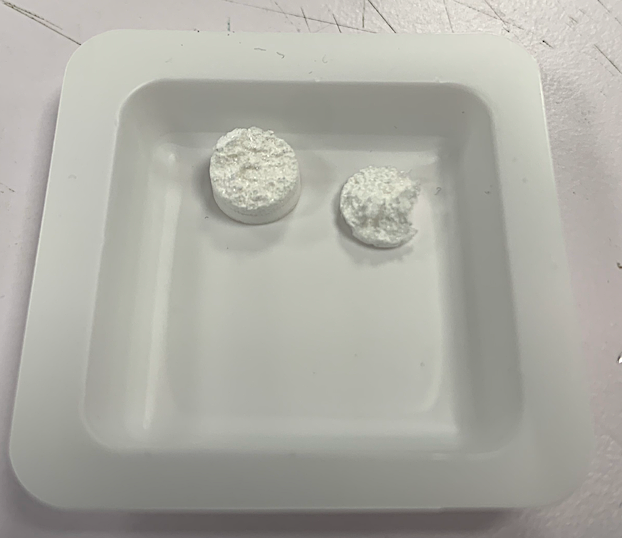 | The crack was observed in the reservoir |
| LF3 | 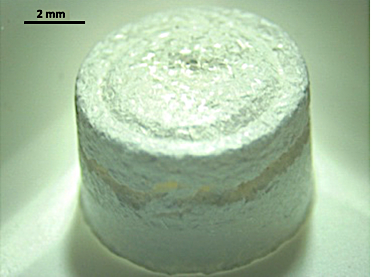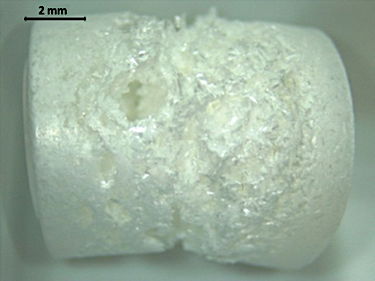 | The crack was observed in the reservoir |
| LF4 | 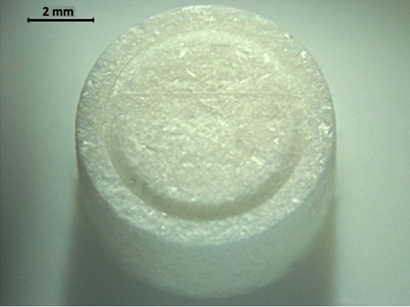 | well-formed, robust, elegant and homogeneous reservoir |
| LF5 | 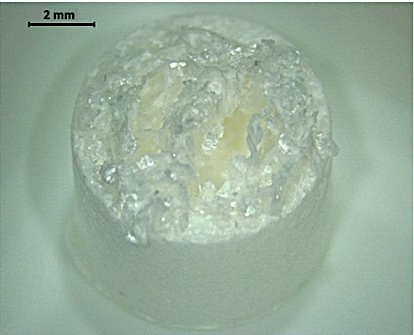 | The crack was observed in the reservoir |
| LF6 | 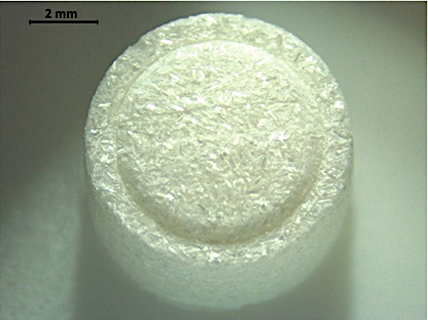 | Well-formed, robust, elegant and homogeneous reservoir |


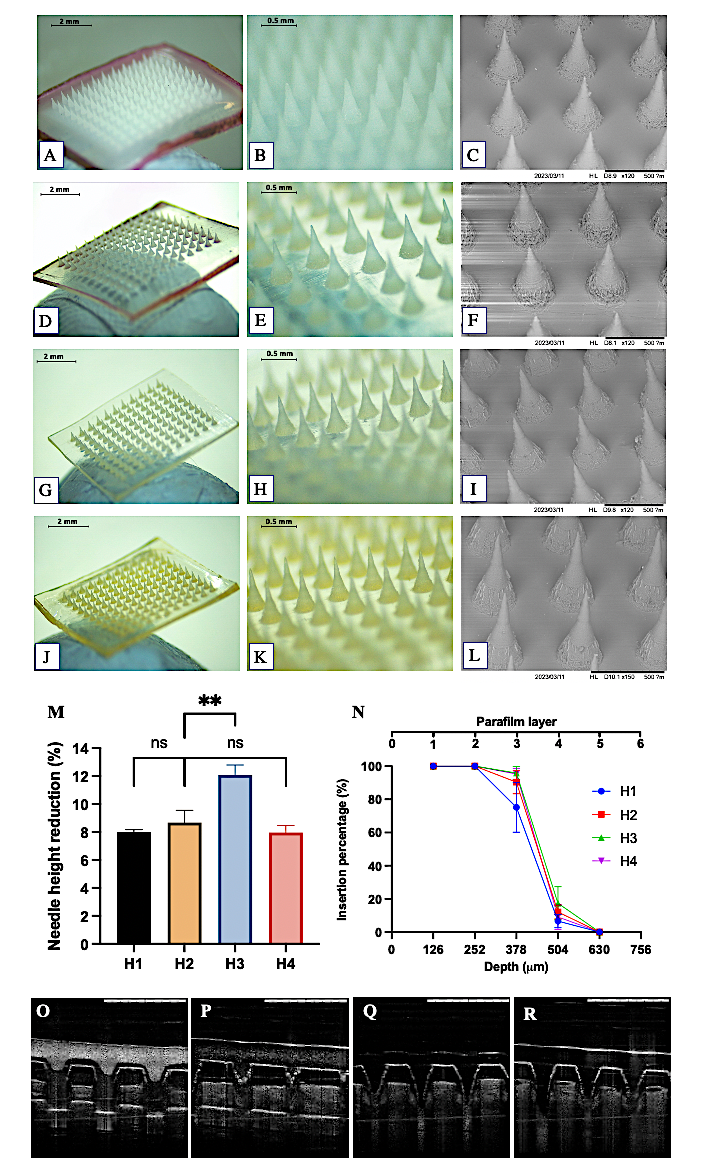


**Fig S1**. Digital images of Gantrez^®^ S-97-based hydrogel-forming MN arrays fabricated from (A, B) H1 and (D, E) H2, and PVA-based hydrogel-forming MN arrays of (G, H) H3 and (J, K) H4. SEM images of MN arrays fabricated from (C) H1, (F) H2, (I) H3 and (L) H4. (M) Percentage height reduction for H1-H4 hydrogel MN arrays after applying a 32 N of force for 30 sec (means + SD., n = 3). (***p*<0.01). (N) Percentage needle insertion for H1-H4 MN arrays in each layer of Parafilm^®^ M after applying a 32 N force for 30 s (means ± SD, n = 3). OCT images of MN arrays fabricated from (O) H1, (P) H2, (Q) H3, (R) H4. The white scale bar represents a length of 1 mm.


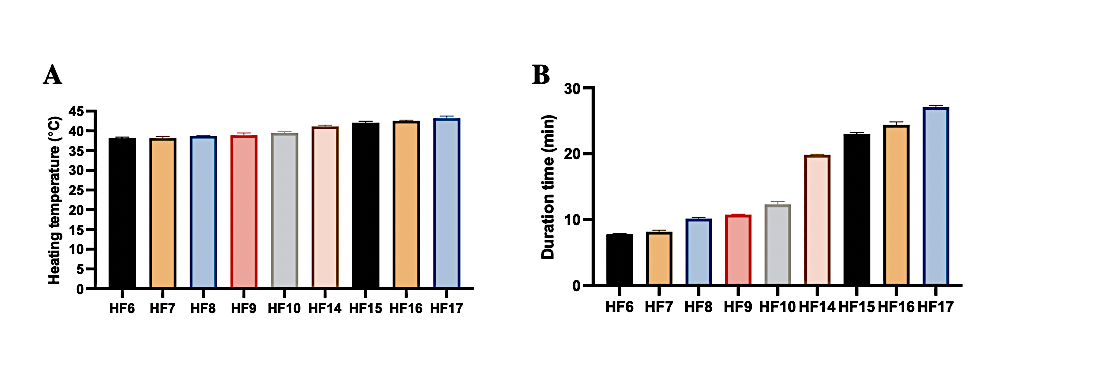


**Fig S2.** (A) the heating temperature (B) the duration of heat-generating powder mixtures (HF6-HF10 and HF14-HF17) (Means + SDs, n=3).

**
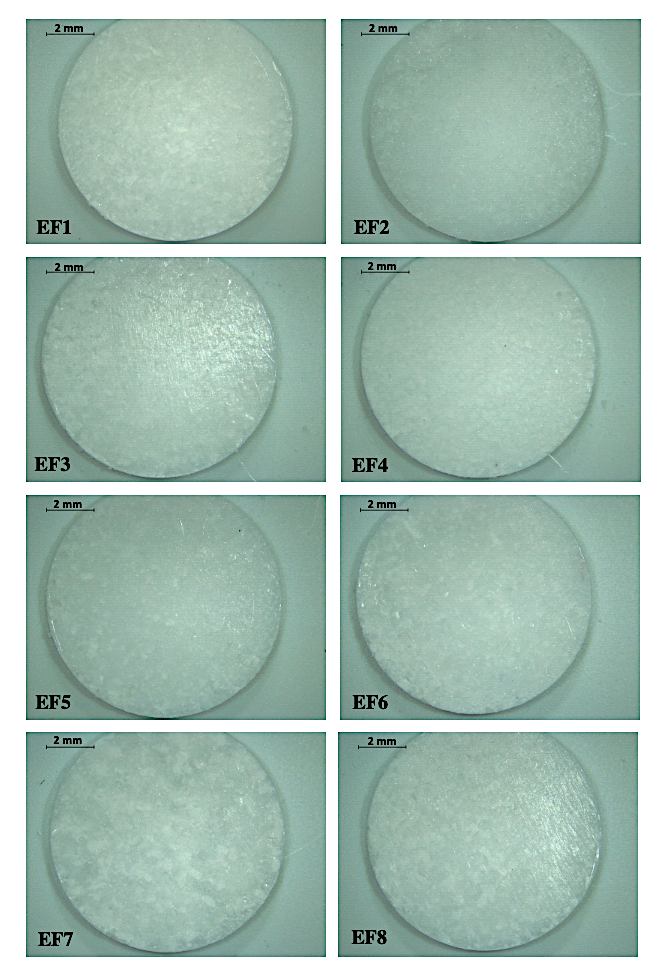
**

**Fig S3.** Microscopic images of EF1-EF8 effervescent reservoir formulations.


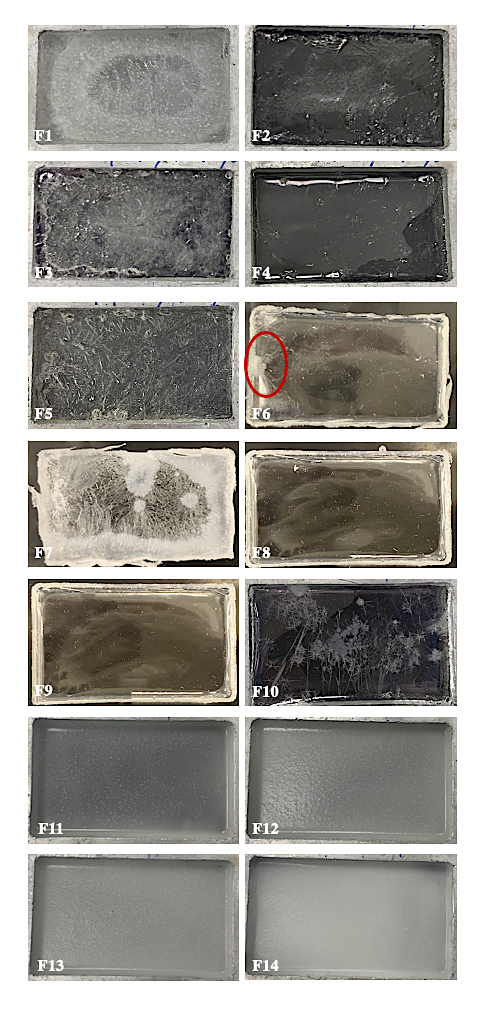


**Fig S4.** Morphology of F1-F14 IBU sodium-loaded polymeric film formulations (30 mm × 50 mm).
